# Intramolecular Triplet–Triplet Annihilation Photon Upconversion in Diffusionally Restricted Anthracene Polymer

**DOI:** 10.1021/acs.jpcb.1c02856

**Published:** 2021-06-03

**Authors:** Fredrik Edhborg, Hakan Bildirir, Pankaj Bharmoria, Kasper Moth-Poulsen, Bo Albinsson

**Affiliations:** Department of Chemistry and Chemical Engineering, Chalmers University of Technology, Gothenburg 412 96, Sweden

## Abstract

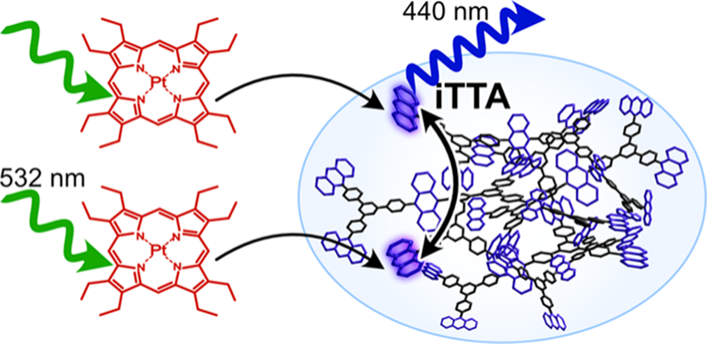

In the strive to develop triplet–triplet annihilation photon upconversion (TTA-UC) to become applicable in a viable technology, there is a need to develop upconversion systems that can function well in solid states. One method to achieve efficient solid-state TTA-UC systems is to replace the intermolecular energy-transfer steps with the corresponding intramolecular transfers, thereby minimizing loss channels involved in chromophore diffusion. Herein, we present a study of photon upconversion by TTA internally within a polymeric annihilator network (iTTA). By the design of the annihilator polymer and the choice of experiment conditions, we isolate upconversion emission governed by iTTA within the annihilator particles and eliminate possible external TTA between separate annihilator particles (xTTA). This approach leads to mechanistic insights into the process of iTTA and makes it possible to explore the upconversion kinetics and performance of a polymeric annihilator. In comparison to a monomeric upconversion system that only functions using xTTA, we show that upconversion in a polymeric annihilator is efficient also at extremely low annihilator concentrations and that the overall kinetics is significantly faster. The presented results show that intramolecular photon upconversion is a versatile concept for the development of highly efficient solid-state photon upconversion materials.

## Introduction

**Figure 1:**
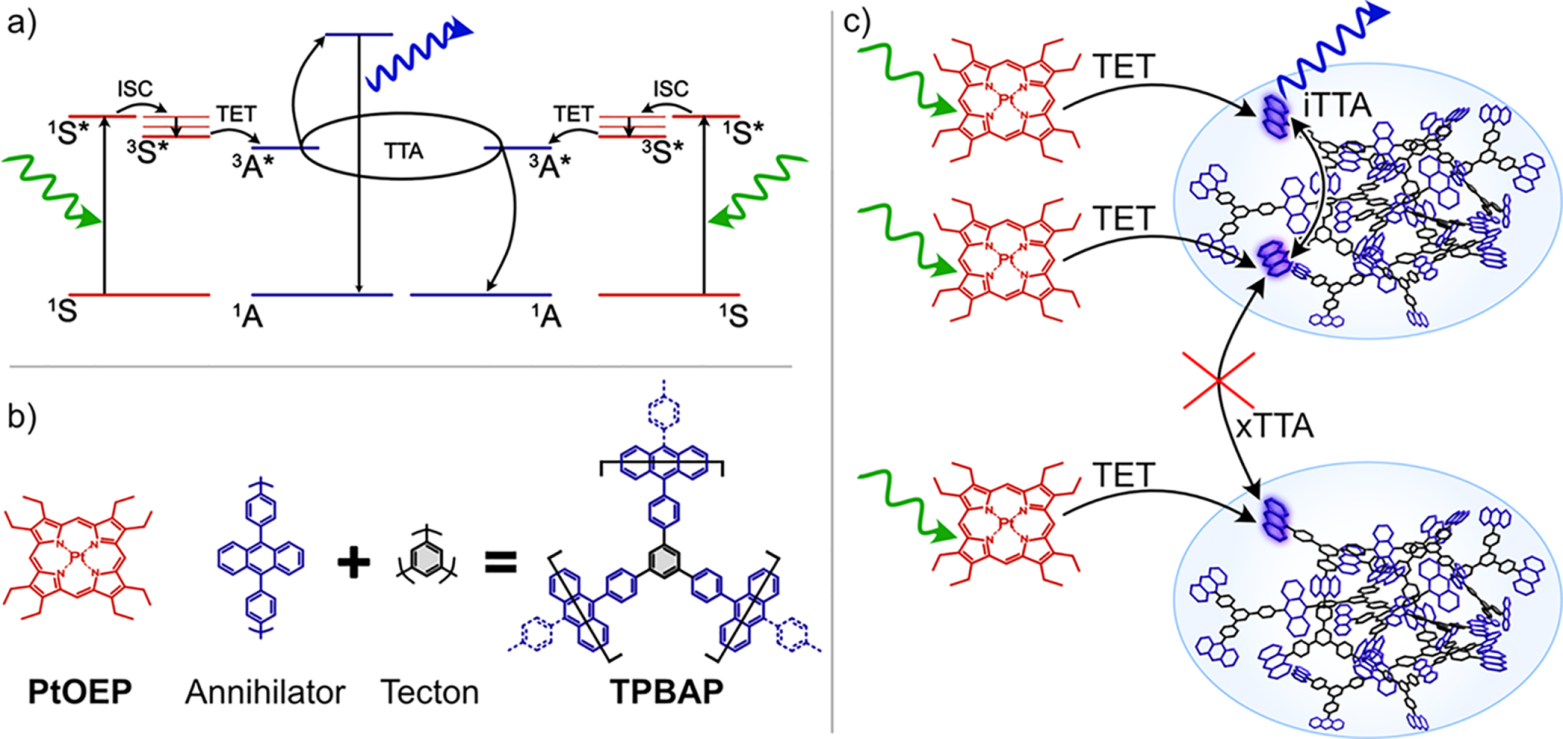
(a) Jablonski diagram describing photon upconversion by TTA. S = sensitizer, A = annihilator, ISC = intersystem crossing, TET = triplet energy transfer, TTA = triplet–triplet annihilation. (b) Molecular structure of the sensitizer **PtOEP** as well as the annihilator **TPBAP** composed of annihilator units sequentially connected with a tecton^35^ (linker). (c) Schematic illustration of photon upconversion via iTTA within an annihilator polymer molecule or particle and xTTA between the annihilator polymer particles.

Photon upconversion by triplet–triplet annihilation (TTA) is a photophysical process where a multicomponent molecular system is used to combine the energy of two photons to generate one photon of higher energy. Research about photon upconversion has gained increasing momentum in the past two decades for its possible applications in, for example, solar energy technologies, where it could increase the solar energy-harvesting efficiency.^1,2^ Photon upconversion by TTA relies on a sequence of excitation energy-transfer events between two molecular components: the sensitizer and the annihilator. The sensitizer absorbs low energy photons, reaching the triplet excited state by intersystem crossing (ISC), and subsequently transfers the excitation energy to the annihilator by triplet energy transfer (TET). Two triplet excited annihilators can then undergo TTA by combining their excitation energy to elevate one of the annihilator molecules to the higher energy singlet excited state, which in turn emits the upconverted emission by fluorescence. The photophysical process is described in the Jablonski diagram in Figure 1a. Photon upconversion has typically been studied in solutions, in diffusion-dependent systems relying on intermolecular energy-transfer events. In this contribution, we investigate intramolecular TTA in an annihilator polymer. In contrast to intermolecular photon upconversion, intramolecular photon upconversion is not dependent on molecular diffusion and can hence potentially be integrated into a solid-state material, which would be advantageous or even necessary for practical applications.

Several approaches for upconversion in solid materials have been reported, for example, various assemblies of sensitizer and annihilator chromophores,^3,4^ metal–organic frameworks,^5−9^ matrix free (neat) films^10−12^ and incorporation of the upconversion chromophores in a gel,^13,14^ a rubbery polymer matrix,^2,15,16^ or semisolid materials^17−19^ that allows some molecular diffusion. However, with truly intramolecular upconversion, the upconversion system could be integrated into a fully solid material. The kinetics of the overall upconversion process in an intramolecular system can be significantly faster than the intermolecular processes, leading to more efficient systems. A fully intramolecular upconversion system would require the sensitizer to be in close proximity to an annihilator moiety for efficient TET, but this has been shown to induce parasitic quenching of the annihilator fluorescence.^20,21^ One alternative to reduce the fluorescence quenching caused by the sensitizer would be to organize the annihilator molecules in a large network in which the triplet excitons can migrate, thereby enabling a longer distance between the sensitizer and the site of TTA.^22,23^ Such a system would in effect replace intermolecular excitation energy transfer dependent on molecular diffusion in solution by intramolecular exciton migration and intramolecular TTA within a molecular framework.

Several different systems for studying intramolecular TTA have been reported, such as annihilator dimers,^24−28^ oligomers/dendrimers,^23^ and polymers.^29−31^ However, most studies of intramolecular TTA are performed in liquid or semiliquid media where the event of TTA could be governed also by molecular diffusion. One challenge with studying intramolecular TTA in annihilator dimers/polymers in liquid systems is the difficulty to distinguish between the intramolecular TTA within an annihilator molecule carrying two triplet excitons and the corresponding process of intermolecular TTA between two separate dimers/polymers carrying one triplet exciton each. True intramolecular TTA in an annihilator dimer/polymer requires at least two triplet excitons being located on the same annihilator molecule. Recently, Ronchi et al. presented a thorough theoretical investigation about photon upconversion in systems where multiple triplet excitons are located within a confined annihilator system.^32^ A confined system could, for instance, be an annihilator polymer where the triplet excitons are confined within the polymer particle. From the statistical analysis of the likelihood of achieving at least two triplet excitons in one such particle, they found that the photon upconversion threshold intensity,^33^ that is, the excitation intensity above which the upconversion system can reach its maximum efficiency,^34^ could be orders of magnitude lower in a confined upconversion system compared to that in a homogeneous bulk system.

In this article, we present an experimental investigation of TTA photon upconversion in a system consisting of a polymeric annihilator, triphenylbenzene-linked anthracene polymer (**TPBAP**), with platinum octaethylporphyrin (**PtOEP**) as the sensitizer. The molecular structure of **TPBAP**, as shown in Figure 1b, is similar to that of a previously published dendrimer annihilator,^23^ but **TPBAP** is a larger and insoluble polymer that can be dispersed in a solvent to form ∼0.5 μm particles. These particles act as slowly diffusing annihilator clusters that are virtually static in comparison to the fast diffusion of the small dissolved **PtOEP** sensitizer molecules. The **TPBAP** particles act as confined annihilator systems which upon triplet sensitization do TTA internally (iTTA) within an annihilator polymer molecule or particle, as illustrated in Figure 1c. However, external TTA between separate annihilator particles (xTTA) is suppressed because of the slow diffusion of the large **TPBAP** particles. With this design, the overall TTA process, which includes triplet exciton migration within the **TPBAP** particle and subsequent iTTA between adjacent annihilator units, mimics the conditions of an intramolecular solid-state upconversion system where no molecular diffusion of the annihilator is possible. We show that iTTA in a polymeric annihilator can be distinguished in the time domain from xTTA and that the overall kinetics of the upconversion process is significantly faster with the studied polymeric annihilator compared to a monomeric system with 9,10-diphenylanthracene (**DPA**) as the annihilator. Furthermore, we show experimentally that the threshold intensity, above which upconversion can reach its maximum efficiency, is significantly lower for **TPBAP** compared to the corresponding monomeric system. With the studied upconversion system, we take one important step toward a fully intramolecular photon upconversion system. The findings presented here show the relevance of developing intramolecular photon upconversion systems as a way to achieve efficient solid-state photon upconversion materials.

## Materials and Methods

**TPBAP** was synthesized via Suzuki-Miyaura polycondensation between 1,3,5-tris(4-bromophenyl)benzene and 9,10-anthracenediboronic acid bis(pinacol) ester. Details about the synthesis as well as N_2_ gas sorption characterization of the **TPBAP** powder is described in the Supporting Information section 1. The photophysical properties and upconversion performance of **TPBAP** were investigated by ultraviolet–visible (UV–vis) absorption spectroscopy, steady-state emission spectroscopy, and time-resolved emission spectroscopy. All UV–vis absorption spectra were recorded on a Varian Cary 50 spectrophotometer. Steady-state emission spectra were recorded on a Varian Eclipse spectrophotometer. For the determination of **TPBAP** fluorescence quantum yield, a Fluorolog FL3–222 (Horiba Jobin Yvon) spectrophotometer was used. For steady-state photon upconversion emission measurements, a home-built setup was used, consisting of Coherent OBIS LS 532 nm laser as the excitation source, a 1681 SPEX monochromator, and a photomultiplier tube (PMT) detector. A 532 nm notch filter was used in front of the monochromator to reduce the scattered excitation light reaching the detector. The fluorescence lifetime of **TPBAP** was measured using time-correlated single photon counting (TCSPC) with a 377 nm laser diode (PicoQuant) as the excitation source and a microchannel plate-PMT (MCP-PMT) detector in an Edinburgh Instruments LifeSpec II. Upconversion kinetics was investigated by ns time-resolved emission spectroscopy using a home-built system. The excitation source was a Spectra-Physics Quanta-Ray ND:YAG laser with a Primoscan OPO, giving pulsed excitation with a pulse duration of approximately 5–10 ns. The excitation wavelength was set to 532 nm. An Oriel Cornerstone 130 monochromator was used in front of a five-stage PMT detector. A 532 nm notch filter was used in front of the monochromator to reduce the scattered excitation light reaching the detector. When it was necessary, a blue transmitting color filter was used in front of the monochromator to prevent any stray light of red **PtOEP** phosphorescence to reach the detector. Corrections of the recorded emission intensity were made to correct for the intensity loss caused by the color filter. All ns time-resolved emission measurements were carried out in 2 mm quarts cuvettes, with excitation laser beam at right angle to the line of detection and the cuvette at approximately 30° to the direction of the excitation light to yield front-face detection. The instrument setup is described schematically in Figure S5. For the preparation of upconversion samples, **TPBAP** powder was suspended in tetrahydrofuran (THF) and sonicated. The dispersion was centrifuged in order to remove larger aggregates, yielding a supernatant with small enough **TPBAP** particles to be colloidally stable for at least a day. The solvent was evaporated, and the **TPBAP** was later redispersed in THF together with **PtOEP** inside a nitrogen-filled glovebox to receive the final upconversion sample. THF was used as solvent for all experiments, unless otherwise stated. For all experiments presented in this article, only freshly prepared samples were used, which were stable for the time of the experiment. The particle size distribution of the dispersed **TPBAP** was estimated by dynamic light scattering (DLS) and atomic force microscopy (AFM), see the Supporting Information Section 3.1. The concentration of **TPBAP** dispersion was estimated from the UV–vis absorption spectrum, assuming that the molar absorptivity of the anthracene subunits of **TPBAP** is the same as the molar absorptivity of **DPA**, see the Supporting Information section 3.2 for details. In this article, all specified concentrations of the **TPBAP** samples refer to the anthracene subunit concentration.

## Results and Discussion

The results and discussion are divided into three sections. The first section presents the synthesis and characterization of **TPBAP** and shows that **TPBAP** can function as an annihilator in TTA photon upconversion. In the second section, the mechanism of TTA (iTTA or xTTA) in **TPBAP** is investigated by studying the kinetics of upconversion using ns time-resolved emission and by comparing the upconversion efficiency of **TPBAP** with the well-known monomeric annihilator **DPA**. In the third section, the time-resolved upconversion emission data are further analyzed to investigate the **TPBAP** upconversion excitation intensity dependence, which is used in a general discussion about the iTTA upconversion performance.

### Synthesis and Characterization of TPBAP

**Figure 2:**
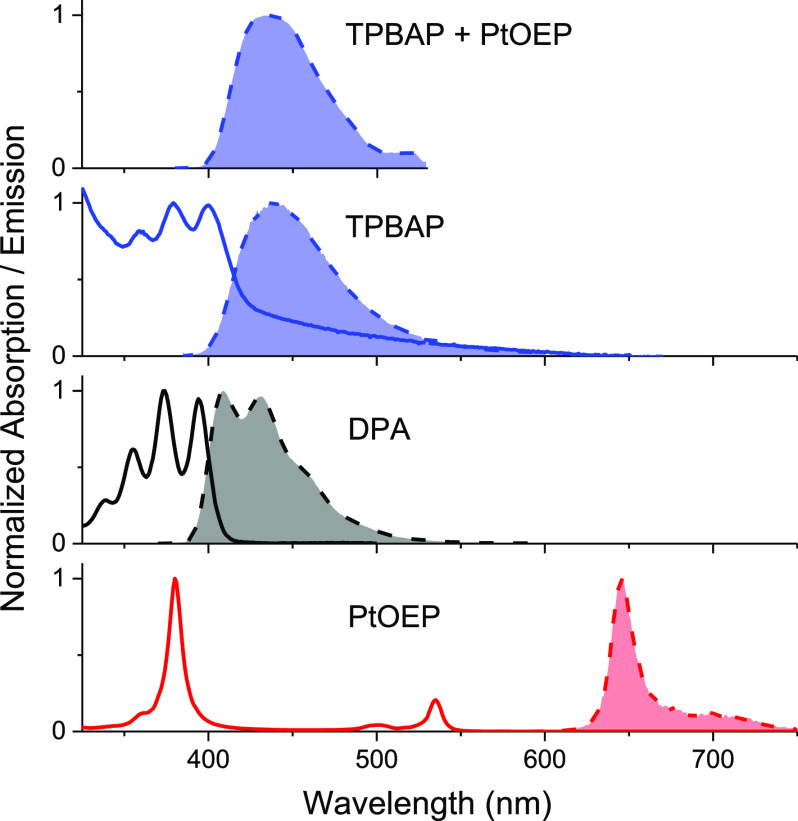
Normalized absorption (solid lines) and emission (dashed lines) spectra of **TPBAP**, **DPA**, and **PtOEP** in THF. Upper panel shows upconversion emission spectrum from **TPBAP** with **PtOEP** as the sensitizer, excited at 532 nm.

**TPBAP** was synthesized with an estimated yield of 66%. The synthesis route through Suzuki-Miyaura polycondensation by coupling a diboronic acid pinacol ester-substituted anthracene with tris(4-bromophenyl)benzene gives a sequential (alternating) cross-linked polymer. This is in contrast to the very similar compound reported by Perego et al. where the cross-linker and anthracene units are randomly copolymerized.^29^ The size distribution of the **TPBAP** particles dispersed in THF was assessed from the DLS analysis, see the Supporting Information section 3.1 for details. The average hydrodynamic diameter of the **TPBAP** particles was found to be 570 ± 30 nm (Figure S6a). The DLS results were further corroborated by AFM imaging of the **TPBAP** dispersion dried on a mica sheet and imaged in the tapping mode, showing particle size in the range similar to that observed from DLS (Figure S6b). The absorption and emission spectra of **TPBAP**, **DPA**, and **PtOEP** are presented in Figure 2. The absorption spectrum of **TPBAP** shows significant light scattering, which is seen as the characteristic unstructured signal increase at shorter wavelengths in the region where the polymer does not absorb. The scattering is caused by the fairly large, hundreds of nanometer-sized, **TPBAP** particles. However, the absorption features of **TPBAP** are similar to its monomeric analogue **DPA**, with the lowest energy absorption band onset around 400 nm and clear vibrational progression into the ultraviolet region. The emission spectrum of **TPBAP** is similar to the **DPA** emission spectrum, but slightly broader and less structured. The fluorescence quantum yield is measured to be approximately 50%, and the amplitude-weighted average fluorescence lifetime is 1.4 ns, see the Supporting Information section 3.3 and 3.4, respectively, for details. Aggregation and sedimentation can be a problem in this type of systems with a nonsoluble annihilator polymer, especially at higher concentrations. However, no notable differences depending on the concentration can be seen in the absorption and emission spectra of **TPBAP** (see Figures S11 and S12), indicating that aggregation/sedimentation does not contribute to any significant errors in the performed analysis. Together, this shows that the anthracene subunits in **TPBAP** act as individual chromophores with photophysical properties similar to those of **DPA**.

Just like **DPA**, **TPBAP** can function as an annihilator in TTA photon upconversion with **PtOEP** as the sensitizer. A spectrum of the upconversion emission can be seen in the upper panel of Figure 2. The upconversion emission spectrum looks the same as the emission spectrum of **TPBAP** except for minor distortions caused by the reabsorption of the sensitizer. A direct comparison of the upconversion emission spectrum and **TPBAP** fluorescence spectrum can be seen in Figure S9. In contrast to **DPA**, **TPBAP** can potentially do TTA internally (iTTA) within the annihilator particle. In order to study the mechanism of TTA in **TPBAP**, an upconversion system with **TPBAP** was designed to favor iTTA over xTTA, using high concentrations of the sensitizer (1 mM) and low concentrations of the annihilator (∼5–30 μM, anthracene subunit concentration). In this UC system, the **PtOEP** sensitizer is dissolved in the solvent surrounding the dispersed **TPBAP** particles, and the triplet sensitization of the annihilator is therefore governed by diffusional collision, as illustrated in Figure 1. One could anticipate that **PtOEP** can also get incorporated into the **TPBAP** network. However, based on the measurements of the **TPBAP** fluorescence lifetime with and without the presence of **PtOEP** together with an estimation of singlet exciton mobility in the **TPBAP** network, it can be concluded that any **PtOEP** incorporation is small and does not affect the conclusions about the mechanism of upconversion (see details in the Supporting Information section 3.4). The unusually high sensitizer concentration and low annihilator concentration is very nonideal in terms of upconversion quantum yield, and this experiment is therefore mainly designed to achieve mechanistic insights.

### Mechanism of TTA

**Figure 3:**
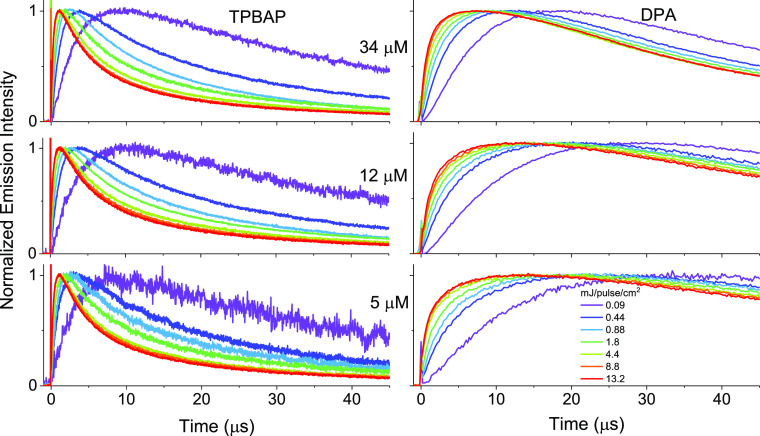
Time traces of normalized upconversion emission intensity for various excitation intensities and annihilator concentrations (excitation wavelength 532 nm, emission wavelength centered at 440 nm). Left: **TPBAP** + **PtOEP**, right: **DPA** + **PtOEP**. Annihilator concentration 34 μM (top), 12 μM (middle), and 5 μM (bottom), **PtOEP** concentration 1 mM. The intense spike at time zero is due to scattered light from the excitation pulse.

To investigate the mechanism of TTA (iTTA or xTTA) in **TPBAP**, the kinetics of the overall upconversion process was analyzed using ns time-resolved emission. To distinguish iTTA from xTTA, the upconversion performance of **TPBAP** was compared to a corresponding upconversion system with **DPA** as the annihilator, which only performs photon upconversion by xTTA, that is, TTA between individually diffusing annihilator units. The fundamental difference between iTTA and xTTA in the studied systems is the number of energy-transfer events that are governed by (slow) molecular diffusion. For the **DPA** system, both the triplet sensitization and the TTA event require molecular diffusion. In contrast, it is only triplet sensitization that requires molecular diffusion for upconversion in **TPBAP** by iTTA. This central difference is hypothesized to be reflected in the upconversion kinetics. Therefore, time-resolved upconversion emission can potentially provide mechanistic information that cannot be obtained only from steady-state measurements. In order to enable direct comparison between the two studied upconversion systems, each measurement of upconversion emission from the **TPBAP** samples was directly followed by the measurement of a reference sample composed of **DPA** and **PtOEP** with the same corresponding (anthracene subunit) concentrations. Figure 3 shows the time traces of upconversion emission at early times from **TPBAP** with various annihilator concentrations and excitation intensities, together with the results for the **DPA** references (full time non-normalized upconversion emission time traces can be seen in Figure S13). First, it can be noticed in Figure 3 that higher excitation intensity results in a faster kinetics of the overall photon upconversion process, both for the **TPBAP** and **DPA** systems. Increasing the excitation intensity is in effect the same as increasing the initial concentration of the triplet excited sensitizer, which gives faster buildup of triplet excited annihilators. Because the triplet sensitization of the annihilator is governed by the diffusion of **PtOEP** molecules in both the upconversion systems, it is expected that both systems show similar dependence on the excitation intensity at early times (see discussion below and Supporting Information section 4.3). Second, from Figure 3, it is evident that the kinetics of the overall photon upconversion process is faster for **TPBAP** as the annihilator compared to the respective **DPA** reference sample. Especially, the rise of the upconversion emission signal for **TPBAP**, which is calculated as the time to half maximum, is 2–5 times faster compared to **DPA** at the respective concentration and excitation intensity. This result cannot be explained with an upconversion mechanism relying only on the diffusion of annihilator molecules because the **TPBAP** annihilator particles are many orders of magnitude larger than the **DPA** molecules and hence diffuse slowly. However, assuming that upconversion is governed by fast iTTA within the **TPBAP** particle but by slower diffusion-limited xTTA for the **DPA** system, this result is expected.

**Figure 4:**
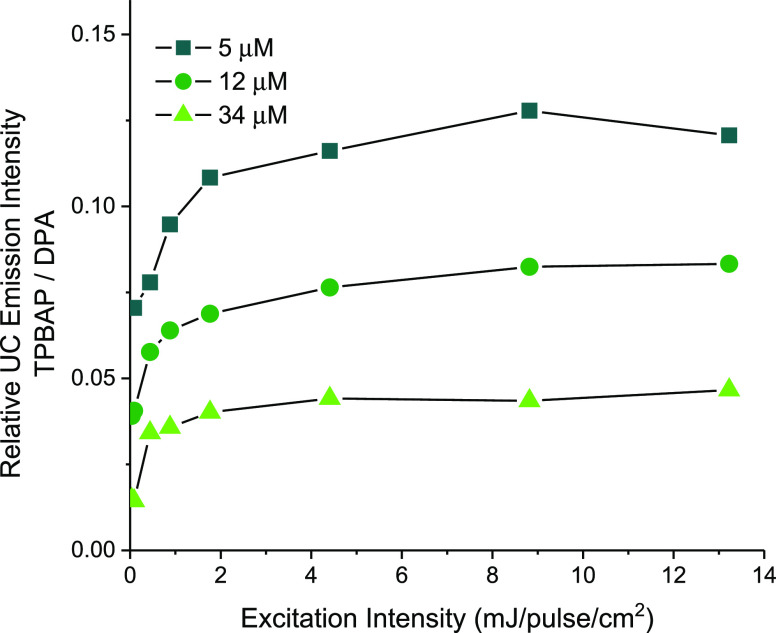
Relative integrated upconversion emission intensity of **TPBAP** + **PtOEP**/**DPA** + **PtOEP** for three different annihilator concentrations (annihilator subunit concentration) and for various excitation intensities. The concentration of **PtOEP** is 1 mM for all the samples. Connecting lines are a guide to the eye.

Even though the upconversion systems of **TPBAP** and **DPA** described in the experiment above are far from ideal in terms of upconversion efficiency, the relative efficiency of the two systems can be used to further elucidate the mechanism of TTA. An upconversion system with monomeric annihilators (such as **DPA)** is highly dependent on the annihilator concentration: at a low annihilator concentration, the upconversion efficiency is low as a result of a poor yield of TET from the sensitizer to the annihilator, as well as a low yield of TTA. In contrast, an upconversion system relying on iTTA in an annihilator polymer is expected to be functional also at extremely low annihilator concentrations because of fast iTTA that is not limited by the diffusion of annihilator molecules. A measure of the upconversion emission intensity can be achieved by integrating the (non-normalized) upconversion emission time traces presented in Figure 3 (full time non-normalized time traces are shown in Figure S13). The integrated upconversion emission after excitation with an intense ∼10 ns laser pulse cannot be directly compared to the upconversion emission in a steady-state experiment with a continuous excitation source;^36^ however, it can be used to investigate the upconversion efficiency of **TPBAP** under varying excitation intensities.^37^Figure 4 shows the upconversion emission intensity of **TPBAP** relative to **DPA**, that is, the time-integrated upconversion emission of **TPBAP** divided by the integrated upconversion emission of **DPA**, for each concentration and excitation intensity. From Figure 4, it is clear that the overall upconversion efficiency of **TPBAP** is low: **TPBAP** only shows 4–12% of the upconversion emission from the corresponding **DPA** sample, depending on the annihilator concentration. However, a clear trend is notable that lower annihilator concentration gives a higher relative upconversion efficiency for **TPBAP**. This finding is in line with what is expected for an annihilator polymer where upconversion is governed by iTTA; diffusion-mediated xTTA in **DPA** suffers more from the long average distance between the annihilator molecules at low concentrations than iTTA within an annihilator polymer, where the annihilator units are always in close proximity. In the extreme limit of an infinitely diluted annihilator, **DPA** would show no upconversion while **TPBAP** could still function as an annihilator.

As evident from Figure 4, **TPBAP** shows a much lower upconversion efficiency than **DPA**. However, to give a more correct picture, this efficiency must be compensated for the differences in the fluorescence quantum yield of the annihilators. **DPA** has a fluorescence quantum yield close to unity in degassed solutions,^38^ which can be compared to a fluorescence quantum yield of approximately 50% for **TPBAP** (see details in Supporting Information section 3.3). Furthermore, the yield of TET from the sensitizer to the annihilator is much higher for **DPA** than that for **TPBAP**. This can be seen from the shorter lifetime of **PtOEP** phosphorescence in the presence of the annihilator, Figure S14, where the **PtOEP** emission is clearly quenched by **DPA**, but insignificantly quenched by **TPBAP**: TET from **PtOEP** to **TPBAP** is so minor that it is not measurable under these experimental conditions. In order to estimate the difference in the triplet sensitization of the annihilator, the rate constant of TET from **PtOEP** to **TPBAP** has been determined in a separate Stern-Volmer experiment, see Supporting Information section 3.6 for details. The rate constant of TET from **PtOEP** to **TPBAP** was determined as *k*_TET_^TPBAP^= 8.2·10^7^ M^–1^ s^–1^, which can be compared to *k*_TET_^DPA^= 2.15·10^9^ M^–1^ s^–1^.^39^ The lower rate constant of TET for **TPBAP**, ∼26 times lower than that for **DPA**, is caused by the slow diffusion of **TPBAP** particles. Also, in a **TPBAP** particle, many of the annihilator subunits are located inside the particle and is therefore not accessible for triplet sensitization from **PtOEP** in the surrounding solution. To summarize, considering the lower fluorescence quantum yield and the much lower rate of TET, the upconversion efficiency of **TPBAP** is surprisingly high compared to **DPA** under these experimental conditions. This finding could be explained by the efficiency of iTTA in **TPBAP** being higher than xTTA in the **DPA** system, which compensates for the lower triplet sensitization and fluorescence quantum yield of **TPBAP**.

### iTTA Upconversion Efficiency

**Figure 5:**
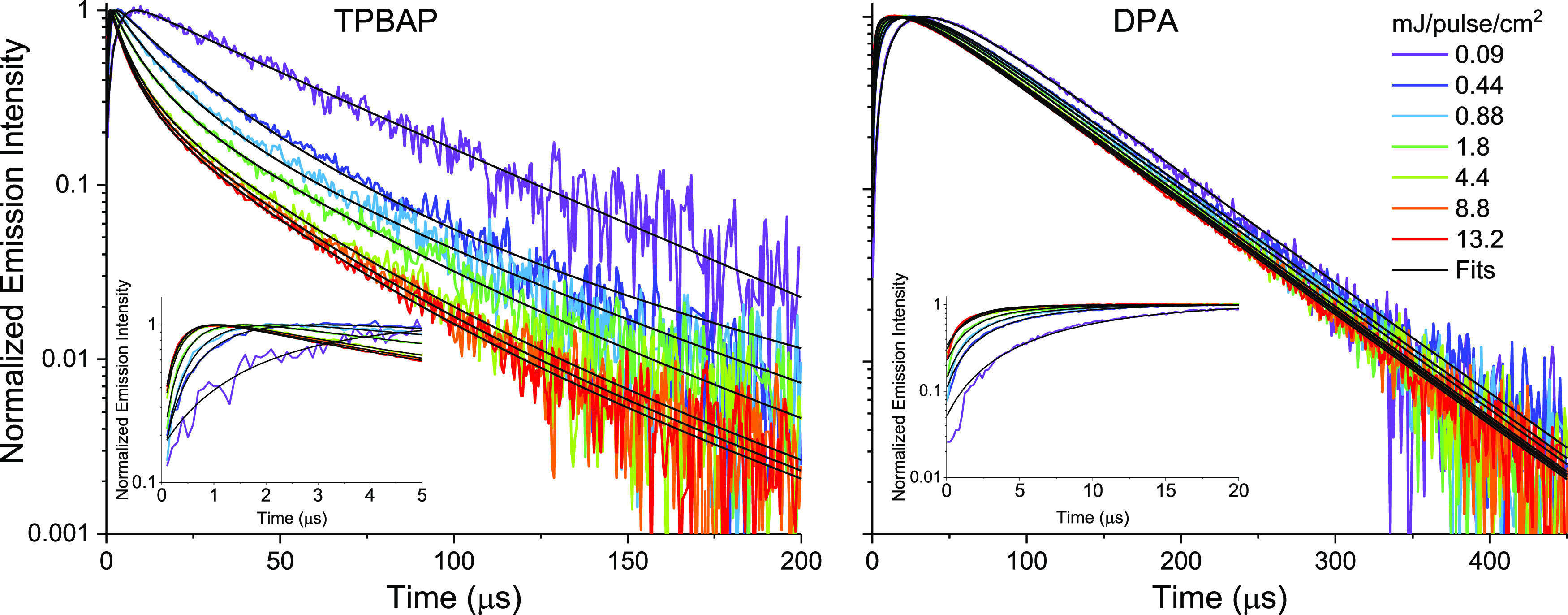
Normalized upconversion emission intensity with fitted time traces for various excitation intensities. Left: **TPBAP** + **PtOEP**, Right: **DPA** + **PtOEP**. Annihilator concentration 5 μM, **PtOEP** concentration 1 mM, excitation wavelength 532 nm, emission wavelength centered at 440 nm. Insets show fit at early times. Data are from the same experiment, as shown in Figure 3. Note the different time scales on the *x*-axis.

Further analysis of the upconversion emission kinetics at longer time scales provides information about the iTTA upconversion performance, efficiency, and excitation intensity dependence. Figure 5 shows time-resolved upconversion emission intensity of **TPBAP + PtOEP** and **DPA + PtOEP** (5 μM annihilator, 1 mM **PtOEP**) in a semilogarithmic plot. The decay profile of the upconversion signal carries information about the deactivation process of the triplet excited annihilators, that is, if it is dominated by spontaneous triplet deactivation (first-order kinetics) or bimolecular TTA (second-order kinetics).

**Figure 6:**
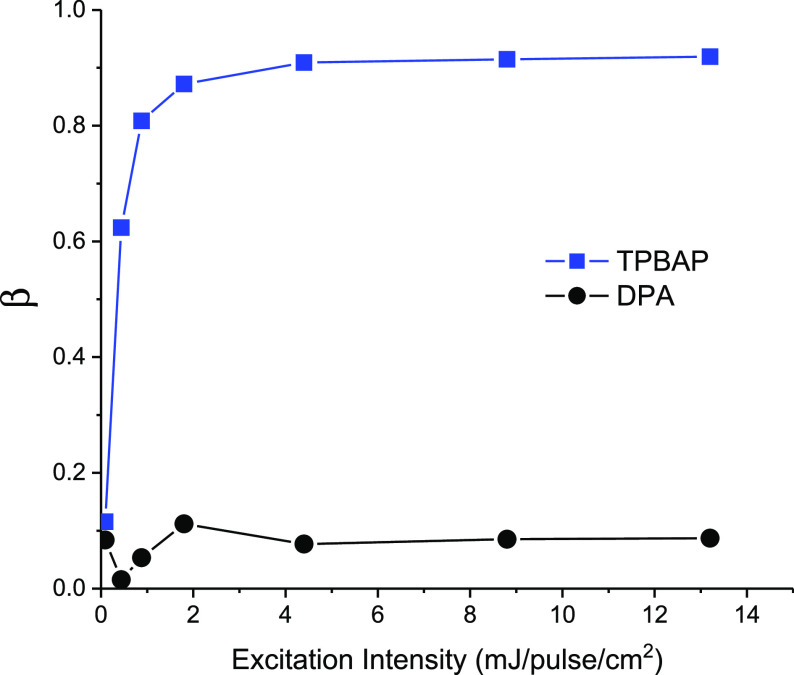
Fitted values of β for **TPBAP** and **DPA** at various excitation intensities. Connecting lines are a guide to the eye. Annihilator concentration 5 μM, **PtOEP** concentration 1 mM, excitation wavelength 532 nm, emission wavelength centered at 440 nm.

From Figure 5, it can be seen that the decay of the upconversion emission from the **DPA** system is monoexponential and almost independent of the excitation intensity, indicating first-order deactivation kinetics of the triplet excited annihilators. In contrast, the decay profile of the **TPBAP** system depends strongly on the excitation intensity and is nonexponential, indicating substantial influence of the second-order triplet deactivation mechanism. The deactivation of a population of triplet excited annihilators can be described by eq 1,
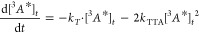
1where [^3^A*]*_t_* is the concentration of the triplet excited annihilator at time *t*, *k_T_* is the rate constant of first-order internal triplet deactivation, and *k*_TTA_ is the rate constant of second-order triplet deactivation by TTA. Equation 1 has an analytical solution shown in eq 2,
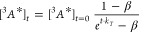
2where [^3^A*]_*t* = 0_ is the initial concentration of the triplet excited annihilator.^34,40^ β, defined in eq 3, is a dimensionless parameter that describes the relative contribution of initial triplet deactivation by TTA.
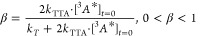
3The rate of TTA, and hence the intensity of upconverted emission, is proportional to the square of the concentration of the triplet excited annihilator.^34,41^ Therefore, the observed time trace of upconversion emission, *I*_UC_(*t*), can be fitted to the function in eq 4.
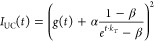
4The function *g*(*t*) is included here as a triplet generating function to take into account that the initial concentration of the triplet excited annihilator in this experiment cannot be described by a simple initial condition. *g*(*t*) is hence a function describing the buildup of the triplet excited annihilator population by triplet sensitization. For the same reason, the initial concentration, [^3^*A* *]_*t* = 0_, shown in eq 2 is replaced by an arbitrary positive coefficient, α, in eq 4. For fitting the observed upconversion emission, the triplet generation function is here arbitrarily chosen to be a sum of two exponential terms, *g*(*t*) = α_1_*e*^–*t* · *k*_1_^ + α_2_*e*^–*t* · *k*_2_^, with α_1_ and α_2_ < 0, in order to obtain a good fit. The results of the fitting are shown as black lines superimposed on the upconversion emission time traces shown in Figure 5. Details about the data fitting and achieved fitting parameters can be found in the Supporting Information section 5. The fitted values of *k_T_* for **TPBAP** and **DPA** are 6.7·10^3^ and 7.3·10^3^ s^–1^, respectively, which gives triplet lifetimes, *τ_T_* = 1/*k_T_*, of 149 and 137 μs, respectively. This shows that the triplet lifetime of **TPBAP** is similar to that of **DPA**. The fitted triplet lifetime of **DPA** is surprisingly short compared to other reported values, which are in the millisecond time range.^24,27,39^ The short fitted triplet lifetime can be explained by the unusually high sensitizer concentration which governs endothermic TET from the annihilator back to the **PtOEP** sensitizer,^21,42,43^ see Supporting Information section 5 for details. The fitted values of β for **TPBAP** and **DPA** at various excitation intensities are shown in Figure 6. For both **TPBAP** and **DPA**, β is approximately 0.1 at the lowest excitation intensity, meaning that triplet decay by TTA is inefficient compared to other deactivation pathways. In contrast to **DPA** for which β stays at this low value, the value of β increases rapidly for **TPBAP** at higher excitation intensities and reaches a plateau at approximately 0.9, which means that triplet decay by TTA is dominating at higher excitation intensities for **TPBAP**. It is expected that the value of β is low at low excitation intensity for **TPBAP** because in a confined annihilator system, the rate of TTA will depend on the likelihood of achieving at least two triplet excitons in the same annihilator particle – at too low excitation intensity, this likelihood is low, giving a lower value of β. The same argument can be used to understand the low relative upconversion efficiency of **TPBAP** at low excitation intensities, as shown in Figure 4. The fitted value of β can be used with eq 3 to estimate an apparent rate constant of iTTA in TPBAP, *k*_iTTA_. The initial concentration of triplet excited annihilators, [^3^*A* *]_*t* = 0_, can be estimated from the measured rate constant of TET from **PtOEP** to **TPBAP** if the initial concentration of the triplet excited sensitizer is known. Under the assumption that the highest excitation intensity used in the experiment is high enough to excite all sensitizer molecules in the excitation volume, (see Supporting Information section 6 for a detail calculation), an apparent rate constant of *k*_iTTA_^TPBAP^ = 1·10^12^ M^–1^ s^–1^ is estimated. In comparison, the same calculations for **DPA** gives *k*_xTTA_^DPA^ = 9·10^9^ M^–1^ s^–1^, which is on the same order of magnitude as the previously reported value of 3.0·10^9^ M^–1^ s^–1^ for xTTA between **DPA** molecules in solutions.^24^ Hence, the apparent rate constant of iTTA within the **TPBAP** network is about two orders of magnitude higher than the diffusion-limited rate constant of xTTA for the corresponding monomeric annihilator. Furthermore, the value of β is related to the upconversion threshold intensity that is often used as a figure of merit for photon upconversion systems. The steady-state threshold intensity, *I*_th_, can be defined as the excitation intensity at which the decay rate of triplet excited annihilators by spontaneous deactivation equals the rate of deactivation by TTA, that is, *k_T_* · [^3^*A* *] = 2*k*_TTA_ · [^3^*A* *]^2^, which gives the point of cross-over from quadratic to linear excitation intensity dependence.^33^ Pulsed excitation results in a similar excitation intensity cross-over point.^34^ The excitation intensity at which β = 0.5 can be regarded as an analogue intensity threshold under pulsed excitation, where β = 0.5 means that the initial rate of triplet deactivation by TTA equals the initial rate of spontaneous triplet deactivation.^40^ As can be seen in Figure 6, the intensity at which β = 0.5 is below 1 mJ/pulse/cm^2^ for **TPBAP**, but a corresponding threshold intensity cannot be estimated for **DPA** because the value of β is below 0.1 also at the highest excitation intensity in the experiment. The much lower threshold intensity for **TPBAP** can be understood in light of the recently reported theoretical work by Ronchi et al.;^32^ an upconversion system where the annihilator triplet excitons are confined in an annihilator network can benefit from the locally high annihilator concentration, which increases the probability of triplet decay by TTA. Similar effect has previously also been shown with a dimeric annihilator.^25^ Furthermore, the cross-over point is very distinct with a clear plateau of the β value at higher excitation intensities. Similar results have previously been observed and explained theoretically by the Monguzzi group,^5^ as a result of the additional criteria for TTA in nondiffusing annihilator particles that TTA can only result from the annihilator particles that are multiple-sensitized and carry at least two triplet excitons. It should be noted that at higher annihilator concentrations, the difference in the β value between the **TPBAP** and **DPA** upconversion system becomes smaller because xTTA in the **DPA** system also becomes efficient at higher annihilator concentrations.

## Conclusions

We have investigated iTTA in an annihilator polymer network. The effects of iTTA were analyzed by comparing the photon upconversion performance and kinetics of the polymeric annihilator with an upconversion system using monomeric **DPA** as an annihilator. The slow diffusion of the polymeric annihilator particles precludes xTTA between the annihilator particles. Therefore, the design of the upconversion system together with the chosen experimental conditions enabled studying the effects of iTTA that otherwise are hard to discern in similar dimeric annihilator systems. This polymer annihilator upconversion system gives a TTA process with triplet exciton migration and subsequent iTTA that mimics conditions in a fully intramolecular upconversion system. We have shown that the overall kinetics of intramolecular photon upconversion is faster than that of upconversion in a corresponding diffusion-controlled monomeric system. Furthermore, we have shown that iTTA can be also efficient at extremely low annihilator concentrations, yielding a much lower threshold intensity compared to a corresponding monomeric upconversion system. These properties could be beneficial in applications where a fast upconversion response is required or low concentrations are necessary, for instance, in imaging systems or biological applications utilizing photon upconversion techniques. The upconversion system studied in this article shows a very low overall upconversion efficiency mainly because of the low yield of TET from the sensitizer to the annihilator and the relatively low fluorescence quantum yield of the annihilator. With a very high sensitizer concentration and very low annihilator concentration used in the experiments presented here, only a small fraction of the sensitizer population will be in a local environment that enables triplet energy transfer to a nearby annihilator, hence the low yield of triplet sensitization. This issue could be resolved by, for instance, chemically attaching the sensitizer to the surface of the annihilator particles, thereby ensuring that all sensitizer molecules in the sample are in close contact with an annihilator, which enables both high rate and high yield of triplet sensitization. Attaching the sensitizer to the surface of a large annihilator particle instead of incorporating it into the annihilator network could also reduce the risk of parasitic singlet energy transfer from a singlet excited annihilator formed by TTA to a nearby sensitizer because triplet exciton migration into the annihilator framework enables larger spatial separation between the site of TTA and the sensitizer.^20,21,44^ Such an upconversion system must be optimized in terms of annihilator particle size and size distribution, where the optimal size depends on the triplet migration length.^32^ Although the upconversion efficiency of the studied system with a polymeric annihilator is low, the results of this study reflect the advantages of intramolecular photon upconversion and its potential for the development of fast and efficient photon upconversion systems in solid-state materials.

## Supporting Information Available

Synthesis and characterization of **TPBAP**; experimental setup of ns time-resolved emission; complementary results of the photophysical investigation of **TPBAP** upconversion; and details from the fitting of upconversion time traces (PDF)
