# Supplementary material for: Intramolecular Triplet–Triplet Annihilation Photon Upconversion in Diffusionally Restricted Anthracene Polymer
Source: J Phys Chem B. 2021 Jun 3;125(23):6255–63. doi: 10.1021/acs.jpcb.1c02856 (PMC8279549; doi:10.1021/acs.jpcb.1c02856)
Supplement: Supplementary file 1 — jp1c02856_si_001.pdf [file jp1c02856_si_001.pdf]

Supporting information

Intramolecular Triplet-Triplet Annihilation Photon Upconversion in Diffusionally Restricted Anthracene Polymer

*Fredrik Edhborg, Hakan Bildirir, Pankaj Bharmoria, Kasper Moth-Poulsen, Bo Albinsson**

Chalmers University of Technology, Department of Chemistry and Chemical Engineering, 412 96 Gothenburg, Sweden.

* Corresponding author: balb@chalmers.se

Contents

1. Synthesis and characterization of TPBAP S2
   1.1 Synthesis S2
   1.2 NMR characterization S3
   1.3 Gas sorption S3
2. ns Time Resolved Emission Experimental Setup S5
3. Characterization of TPBAP dispersion S6
   3.1 Particle size distribution S6
   3.2 Estimation of TPBAP concentration S7
   3.3 Fluorescence quantum yield S7
   3.4 PtOEP incorporation in TPBAP and TPBAP fluorescence lifetime S8
   3.5 Photon upconversion emission S9
   3.6 Rate of triplet sensitization S10
4. Complementary results for time resolved photon upconversion experiments S12
   4.1 Absorption and emission spectrum of TPBAP S12
   4.2 Time resolved upconversion emission raw data S14
   4.3 PtOEP phosphorescence quenching by TPBAP and DPA S14
5. Fitting of upconversion time traces S17
6. Estimating rate constant of iTTA S18
References S19

1. Synthesis and characterization of TPBAP

This section describes synthesis of **TPBAP** and characterization of the raw, as synthesized, solid state **TPBAP** powder.

1.1 Synthesis

TPBAP was synthesized via Suzuki polycondensation between 1,3,5-Tris(4-bromophenyl)benzene **(1)** and 9,10-Anthracenediboronic acid bis(pinacol) ester **(2)** similar to the published procedures (Figure S1).[1,2]

9,10-Anthracenediboronic acid bis(pinacol) ester, 1,3,5-Tris(4-bromophenyl)benzene, tetrakis(triphenylphosphine)palladium(0), 99% potassium carbonate (K_2CO_3), 99.9% anhydrous Sure/Seal THF were purchased from Sigma Aldrich and 2-(9-Anthryl)-4,4,5,5-tetramethyl-1,3,2-dioxaborolane was received from TCI Chemicals. HPLC grade methanol (MeOH) and chloroform ($CHCl_3$) were bought from Fisher Scientific.

0.17 g (0.46 mmol) 1,3,5-Tris(4-bromophenyl)benzene (**1**), 0.2 g (0.31mmol) 9,10-Anthracenediboronic acid bis(pinacol) ester (**2**), and 0.036 g (0.031 mmol) (tetrakis(triphenylphosphine)palladium(0) were put in a 100 mL Schlenk flask, and purged with nitrogen for 30 minutes. In following, 15 mL anhydrous THF and 2.3 mL 2 M aqueous K_2CO_3 solution were added to the reaction mixture, which was subsequently heated to 85°C under inert atmosphere. After stirring for 3 days, the flask was cooled down to room temperature, and 5 mL solution of 0.42 g (1.1 mmol) 2-(9-Anthryl)-4,4,5,5-tetramethyl-1,3,2-dioxaborolane in anhydrous THF was introduced to the media by using a syringe under inert atmosphere. The final mixture was heated to 85°C to be further stirred for 2 hours. The precipitate was filtered, and washed with 100 mL THF, 100 mL MeOH, 100 mL distilled water, 50 mL MeOH, 100 mL $CHCl_3$, 50 mL MeOH, respectively. The collected filtrate was further purified by using the Soxhlet extraction method in MeOH for a day. The collected 0.12 g yellow-orange solid, **TPBAP**, yielded in 66 %.

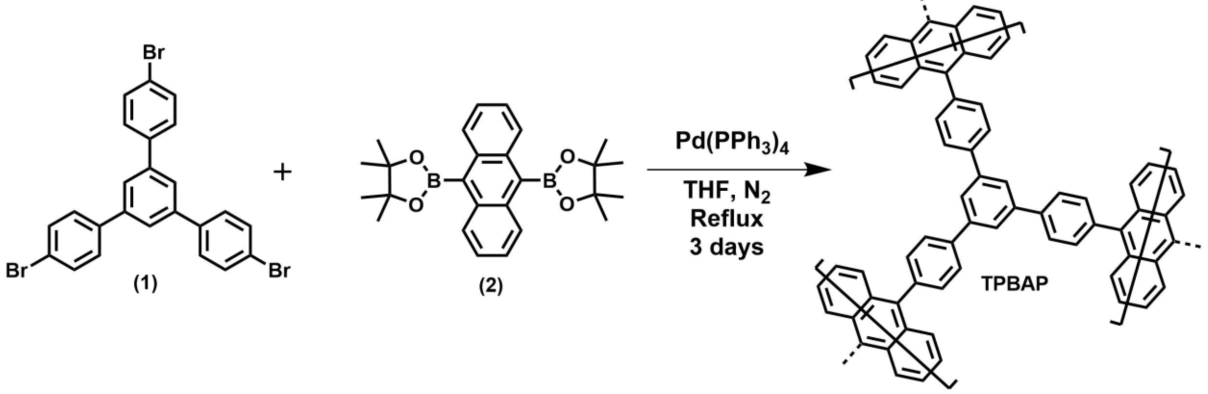

Figure S1. Synthesis route for **TPBAP**.

1.2 NMR characterization

Solid-state cross-polarization magic angle spinning (CP-MAS) ^{13}C nuclear magnetic resonance (NMR) spectra were recorded on Bruker 400 MHz DNP NMR. The NMR spectrum of **TPBAP** can be seen in Figure S2. The broad signals in the aromatic region between 140-120 ppm revealed its fully unsaturated, sp^2 hybridized carbon-based structure.

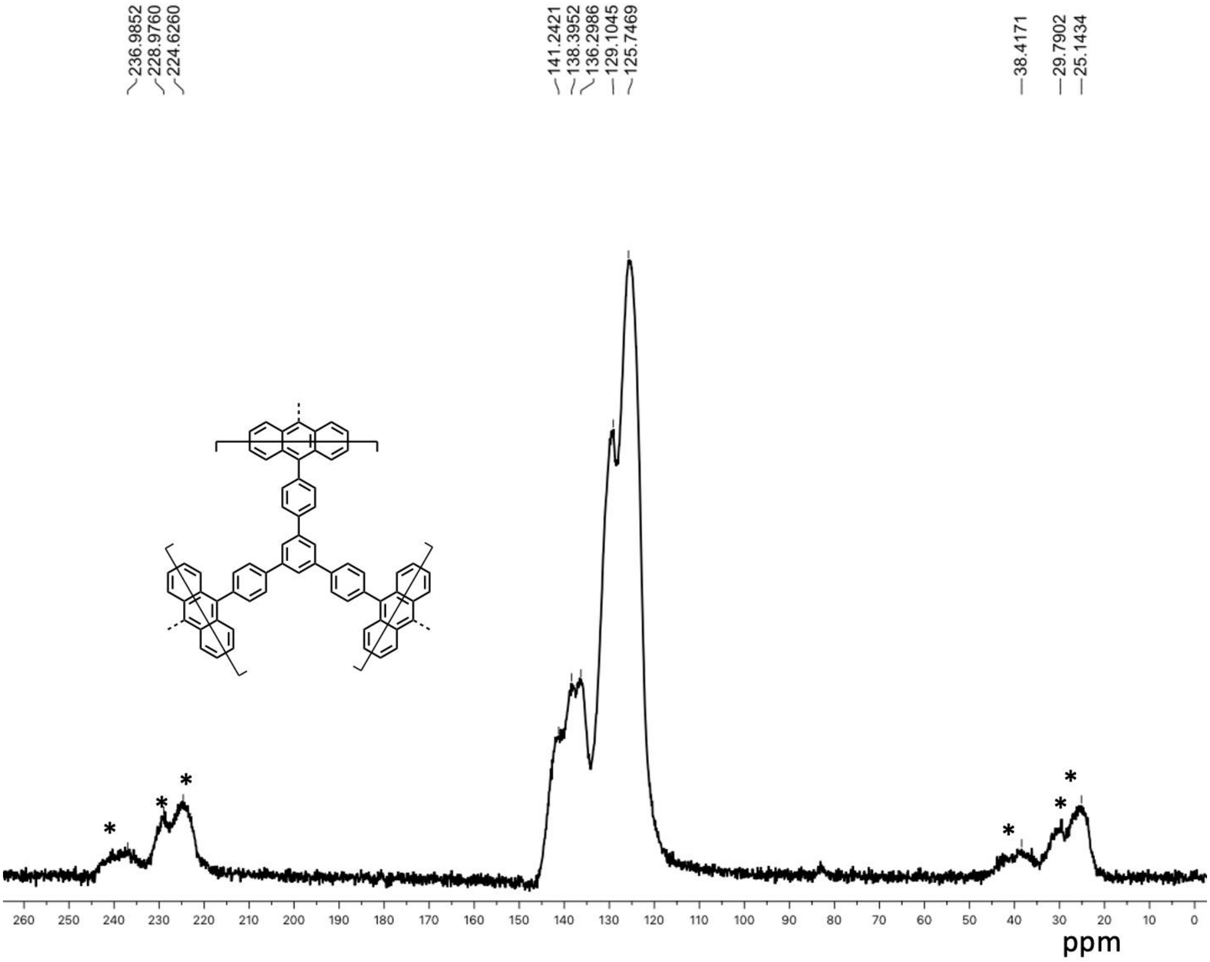

Figure S2. ^{13}C NMR spectrum of **TPBAP**.

1.3 Gas sorption

The specific surface area of **TPBAP** was measured using N_2 gas sorption measurement via the Brunauer–Emmett–Teller (BET) method using Micromeritics TriStar 3000. The result is presented in Figure S3 and the specific surface area was calculated to 511 m^2/g. The steep uptake in the low relative pressures region addressed a Type I isotherm indicating a micropore rich backbone along the polymeric network.[3] Given that, the slight H4 type hysteresis pointed to presence of some small mesopores,[3] which can also be seen from the NL-DFT pore-size distribution (Figure S4) predicting those around 2.3 and 3.1 nm along with the dominant (micro)pores at 1.8 nm. It should be noted that the N_2 gas sorption measurements were made on the as synthesized solid **TPBAP** powder. Therefore, these results do not necessarily represent the surface area or pore-size distribution of **TPBAP** in the upconversion samples where **TPBAP** is dispersed in THF.

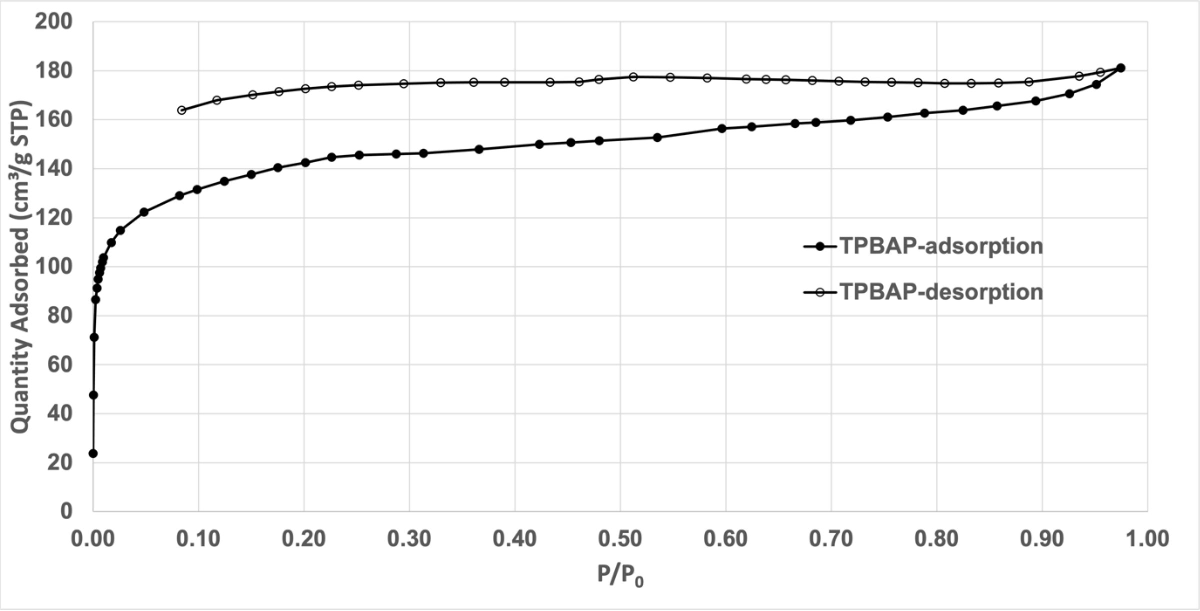

Figure S3. N2 gas sorption isotherm of **TPBAP**.

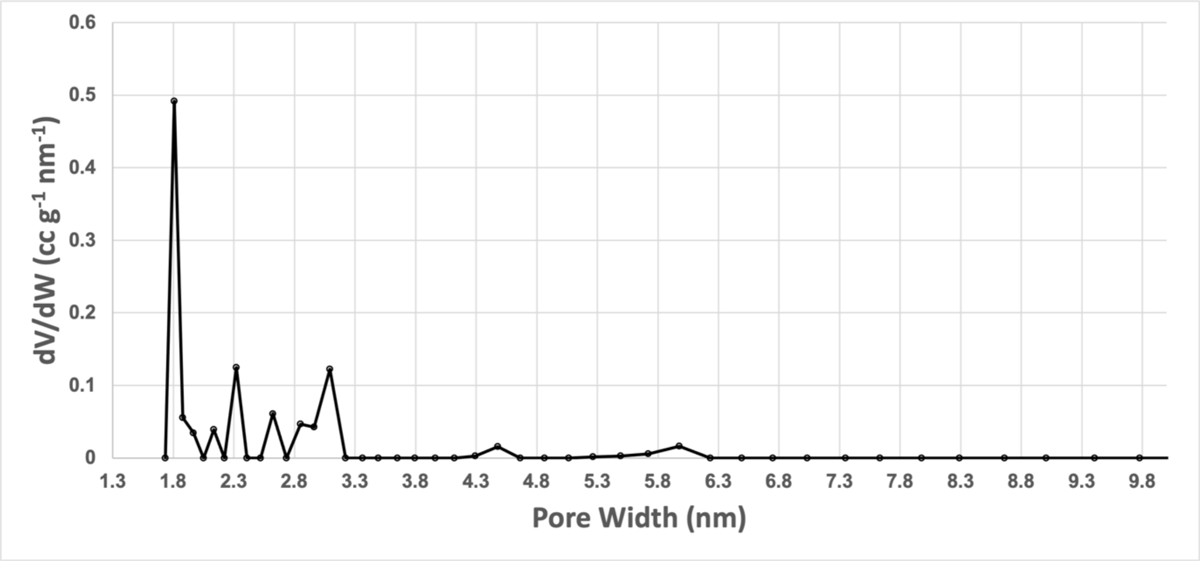

Figure S4. NL-DFT pore-size distribution of **TPBAP**.

2. ns Time Resolved Emission Experimental Setup

Figure S5 shows a schematic illustration of the experimental setup used for ns time resolved upconversion emission measurements. The sample in a 2x10 mm cuvette was positioned at approximately 30° to the excitation laser to yield front face detection. The 532 nm notch filter and blue transmitting color filter was used to reduce the scattered excitation light and emission from the **PtOEP** sensitizer, respectively.

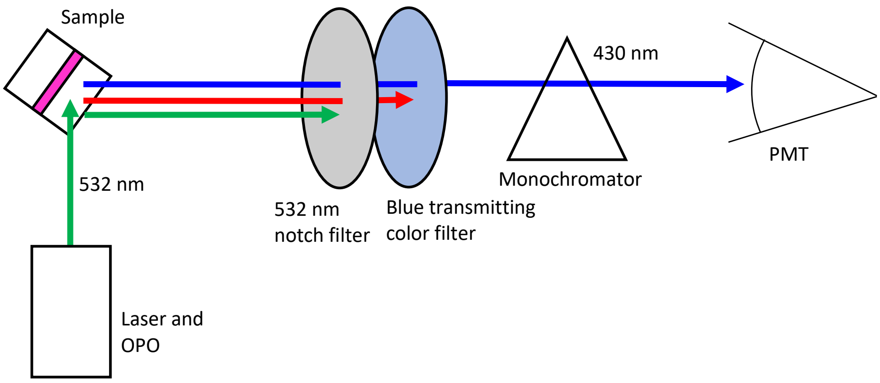

Figure S5. Schematic illustration of ns time resolved emission setup.

3. Characterization of TPBAP dispersion

This section describes morphological and photophysical characterization of **TPBAP** dispersed in tetrahydrofuran (THF), *i.e.* as prepared for the upconversion experiments.

3.1 Particle size distribution

The **TPBAP** particle size distribution was characterized using dynamic light scattering (DLS) and atomic force microscopy (AFM).

Dynamic Light Scattering: Dynamic light scattering (DLS) were performed on a Zeta-Sizer Nanoseries instrument (Malvern Instruments) equipped with built-in temperature controller having an accuracy of ±0.1 K. For size measurements, the samples were contained in a 4 mm path length quartz cuvette and measured at a scattering angle of 173°. An average of 90 measurements were taken for size measurement. The measurements were triplicated to ascertain the accuracy of size obtained. The hydrodynamic diameter was estimated to 570±30 nm, the particle size distribution can be seen in Figure S6a.

Atomic Force Microscopy: The AFM imaging was performed on a Digital Instrument Dimension 3000 Large sample AFM with a type G scanner. A MicroMasch silicon SPM Probe was used for analysis in tapping mode, which was carried out in air. A pyramidal silicon tip with radius of 8 nm, height of 15 µm, cone angle of 40° was used at resonance frequency of 325 kHz and force constant of 40 Nm^{-1}. The sample was prepared by drop casting of a 5 µl **TPBAP** supernatant solution on a thin mica sheet followed by air drying for 30 minutes. Before drop casting the **TPBAP** solution was sonicated and allowed to stand for 15 min. The resulting AFM image can be seen in Figure S6b. The imaging was duplicated for two different sample on different days to ascertain the accuracy of image obtained.

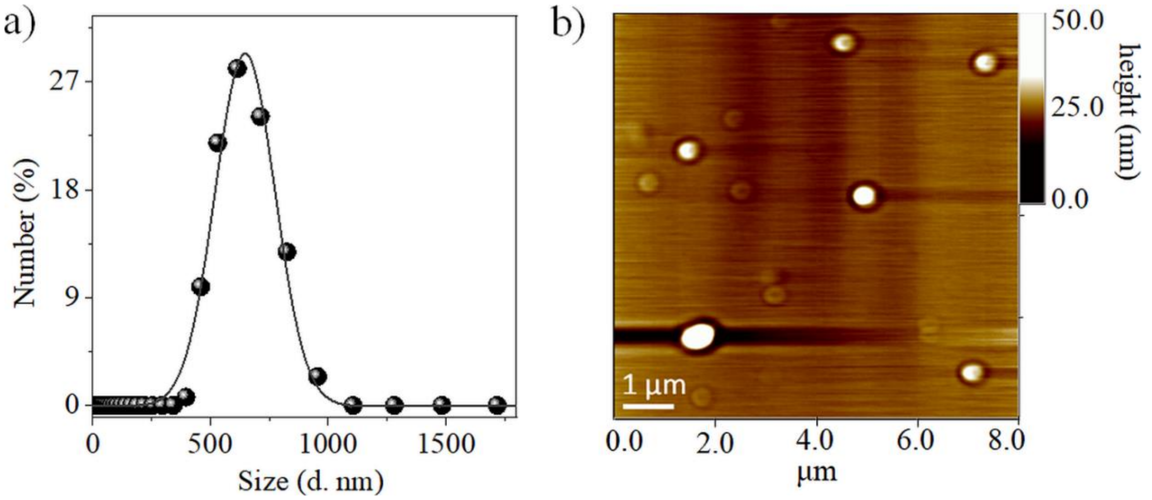

Figure S6. a) Hydrodynamic diameter profile of **TPBAP** particles in THF measured by DLS. b) AFM image of **TPBAP** particles.

3.2 Estimation of TPBAP concentration

The concentration of **TPBAP** dispersed in THF was determined from the absorption spectrum using Lambert-Beer law, $A = \varepsilon c l$. The molar absorptivity, ε, of **TPBAP** was assumed to be the same as the molar absorptivity of **DPA** (per anthracene subunit) at their respective maximum absorption wavelength around 400 nm, 12500 $M^{-1}cm^{-1}$. The non-soluble **TPBAP** particles give rise to significant scattering in the absorption spectrum, seen as an increasing absorption signal at lower wavelengths. The scattering signal was subtracted from the recorded absorption spectrum so that only the true absorption signal of **TPBAP** was used to calculate the concentration. The magnitude of scattering was estimated by drawing a straight line in the absorption spectrum from 445 nm, extrapolating the scattering signal into the UV region, see example in Figure S7. All concentrations of **TPBAP** given in this article refers to the anthracene subunit concentration.

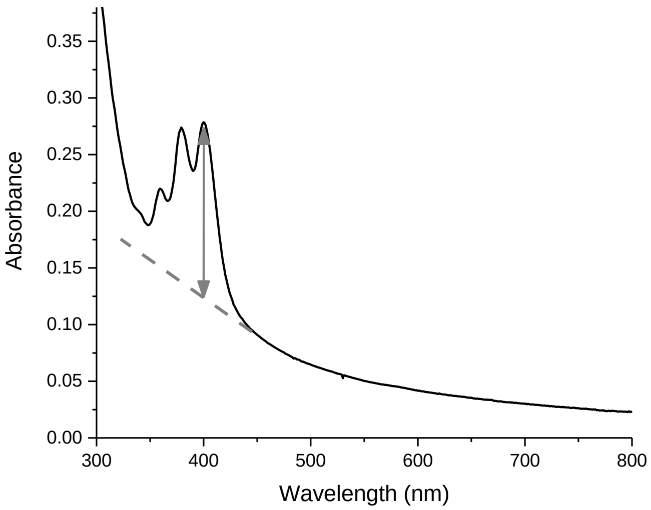

Figure S7. Absorption spectrum of **TPBAP**. Gray dashed line illustrates how the scattering magnitude was estimated. The solid gray arrow was used as a measure of the absorbance of **TPBAP**.

3.3 Fluorescence quantum yield

Fluorescence quantum yield of **TPBAP** in THF was estimated using the relative method with coumarin 480 in EtOH with a fluorescence quantum yield of 0.78 as reference. The fluorescence quantum yield, Φ, was calculated according to Equation S1,

$$\Phi = \Phi_r \frac{(1-10^{-A_r}) \cdot I \cdot \eta^2}{(1-10^{-A}) \cdot I_r \cdot \eta_r^2} \qquad \text{(S1)}$$

where A is the absorption of the sample at the excitation wavelength, I is the integrated emission and η is the refractive index of the solvent[4]. Subscript r refers to the reference sample. The absorption of the **TPBAP** was corrected for scattering as described in Figure S7. The fluorescence quantum yield of **TPBAP** was estimated to 50 $\pm$ 6 % as mean $\pm$ standard deviation of four measurements at different excitation wavelength. The main source of error in

this analysis is likely to be the estimation of absorbance of the **TPBAP** sample due to the scattering.

3.4 PtOEP incorporation in TPBAP and TPBAP fluorescence lifetime

Triplet sensitization of the **TPBAP** annihilator is in this work assumed to be governed by triplet energy transfer from **PtOEP** sensitizer molecules diffusing in the solution surrounding the **TPBAP** particles and not from **PtOEP** molecules incorporated into the **TBPAB** framework. The conclusion that **PtOEP** is not incorporated into the **TPBAP** particles is based on two facts:

First, if the **PtOEP** sensitizer would have been incorporated into the **TPBAP** annihilator network, the upconversion process would be totally independent on molecular diffusion and the resulting upconversion kinetics would likely be on a timescale much faster (<ns) than what was observed (~µs, Figure 3 in the main article).

Second, any significant incorporation of **PtOEP** into the **TPBAP** particles would result in quenching of the **TPBAP** fluorescence[5,6]. Figure S8 shows prompt fluorescence decay upon direct excitation of **TPBAP** dispersed in THF with and without presence of 1 mM **PtOEP**, measured by time correlated single photon counting. Table S1 shows the fitted fluorescence lifetime resulting from a biexponential fit with deconvolution of the instrument response function (IRF). The fluorescence decay kinetics is unaffected by presence of **PtOEP**, which shows that the singlet excited state of **TPBAP** is not quenched by **PtOEP** at the studied concentrations. It could be argued that that the spatial separation between a localized **TPBAP** singlet exciton and any incorporated **PtOEP** molecule is too large to yield any observable quenching. However, the **TPBAP** singlet exciton is likely to be highly mobile in the **TPBAP** network, which increases the likelihood of quenching by **PtOEP**. A rough estimate of the singlet exciton mobility can be given from the Förster resonance energy transfer (FRET) theory, from which the rate of energy transfer from one anthracene unit to its nearest neighbor can be calculated. Assuming a spectral overlap integral similar as for **DPA** (J= $2.4 \cdot 10^{13}$ nm^4/M cm) (ref. 7), orientation factor for meta coupled phenyl anthracenes, $\kappa^2 = 3.06$ (ref. 7) and anthracene-anthracene distance of 13 Å, the FRET distance, R_0, can be calculated to 48 Å and the FRET rate constant, k_{FRET}, to $1.8 \cdot 10^{12}$ s^{-1} (ref. 7). This gives a time constant of FRET $\tau_{FRET} = 1/k_{FRET}$ = $5.2 \cdot 10^{-13}$ s. This means that the time constant for a localized **TPBAP** singlet exciton to jump from one anthracene unit to another is on the ps timescale. Therefore, it is plausible that a singlet exciton has time to migrate through large sections of a **TPABP** molecule within its excited state lifetime of 1.4 ns. It is also likely that the FRET theory underestimates the singlet exciton mobility because it only considers the through-space dipole-dipole interactions and does not take any through-bond interactions into account.

Based on the discussion above it can be concluded that any **PtOEP** incorporation into the **TPBAP** framework is very small and does not affect the mechanistic description of the observed upconversion emission.

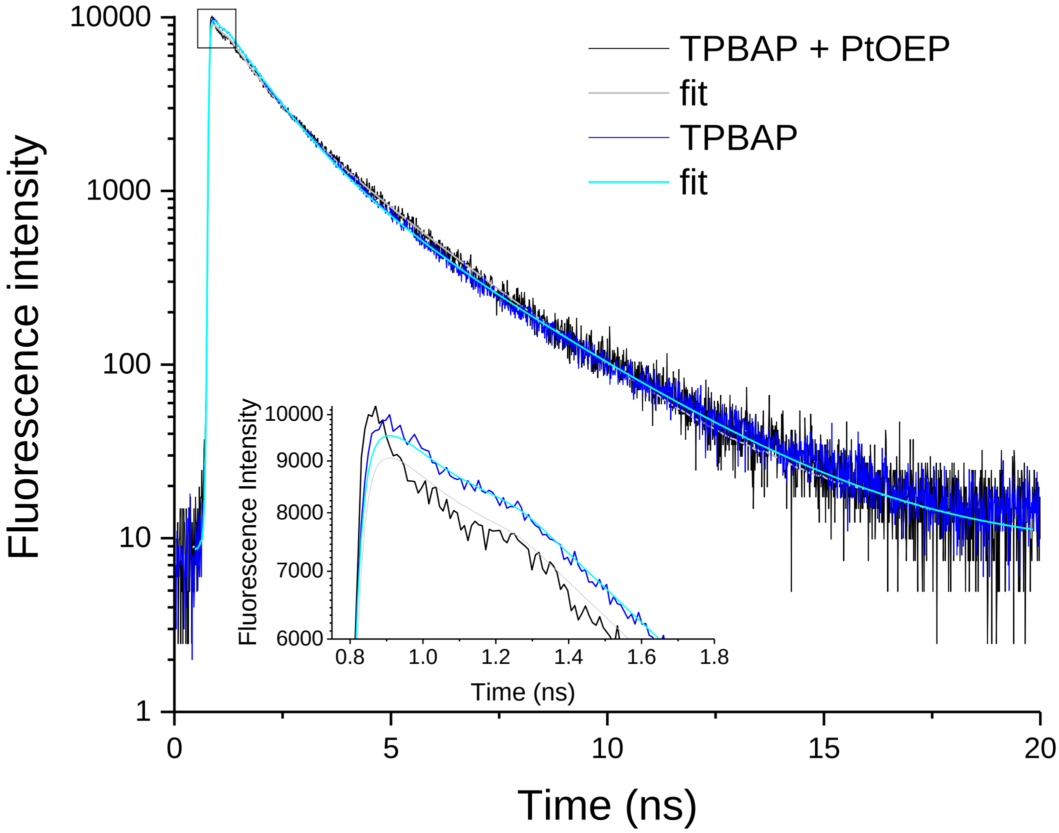

Figure S8. Fluorescence decay of direct excited **TPBAP** (5 µM anthracene subunit concentration) in air equilibrated THF with (black trace) and without (blue trace) 1 mM **PtOEP**. Gray and cyan lines show fits (biexponential decay with IRF deconvolution) to the respective time trace. Excitation wavelength 377 nm, emission wavelength centered at 440 nm.

Table S1. Fitted lifetimes, τ, and amplitudes, A, of fluorescence decay traces in Figure S8.

	$\boldsymbol{\tau_1}$ (ns)	$\boldsymbol{\tau_2}$ (ns)	$\boldsymbol{A_1}$ (a.u.)	$\boldsymbol{A_2}$ (a.u.)	$\boldsymbol{<\tau>}$ [a] (ns)
**TPBAP**	2.75	0.98	2619	9259	1.37
**TPBAP + PtOEP**	2.30	0.77	1893	2712	1.40

[a] Amplitude weighted average lifetime. $<\tau> = (A_1\tau_1 + A_2\tau_2)/(A_1 + A_2)$

3.5 Photon upconversion emission

Photon upconversion emission spectrum of **TPBAP** with **PtOEP** sensitizer can be seen in Figure S9 together with fluorescence spectrum of directly excited **TPBAP**. The difference

between the upconversion and direct excitation emission spectrum is likely caused by reabsorption of the upconversion emission by the sensitizer.

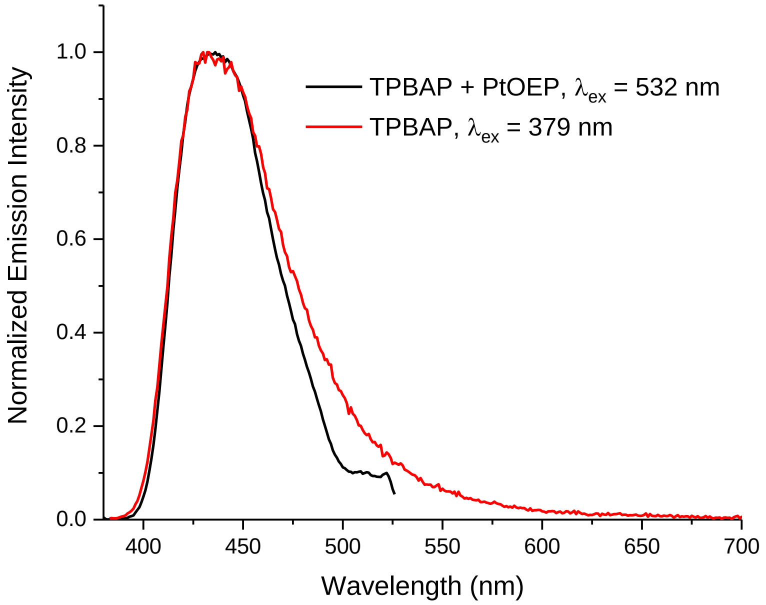

Figure S9. Black line: Upconversion emission spectrum of **TPBAP** (3 μM) + **PtOEP** (1 mM) in THF, excitation wavelength 532 nm. Red line: Emission spectrum of **TPBAP** (3 μM) in THF, excitation wavelength 379 nm.

3.6 Rate of triplet sensitization

The rate constant of triplet energy transfer, k_{TET}, from **PtOEP** to **TPBAP** have been determined by measuring the quenching of **PtOEP** phosphorescence in presence of **TPBAP**. Phosphorescence lifetime of **PtOEP** in THF was measured using a Cary Eclipse fluorometer and the emission time trace was fitted to mono exponential decay, see Figure S10. According to Equation S2, the ratio of **PtOEP** phosphorescence lifetimes without and with **TPBAP**, τ_0/τ, is proportional to concentration of the quencher, $[\boldsymbol{TPBAP}]$, with $k_{TET} \cdot \tau_0$ as proportionality constant. Using this relationship, k_{TET} can be calculated from the slope of the linear fit in the Stern-Volmer plot in Figure S10. The fitted slope is 7979 M^{-1}, which gives k_{TET} = 8.2 · 10^7 $M^{-1}s^{-1}$ using τ_0 = 97.6 μs.

$$\frac{\tau_0}{\tau} = 1 + k_{TET} \cdot \tau_0 \cdot [TPBAP] \qquad \text{(S2)}$$

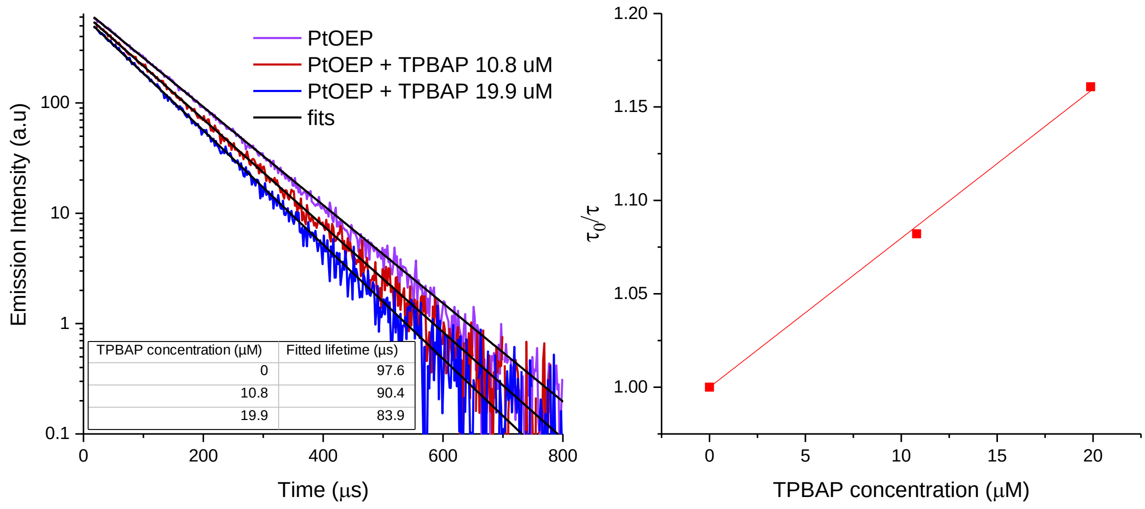

Figure S10. Left: Phosphorescence decay of **PtOEP** and **PtOEP+TPBAP** in THF. **PtOEP** concentration 10 uM, excitation wavelength 535 nm, emission wavelength 646 nm. Inset table shows fitted lifetime of the respective decay (monoexponential fit). Right: Stern-Volmer plot of relative phosphorescence lifetimes versus **TPBAP** concentration (anthracene subunit concentration).

4. Complementary results for time resolved photon upconversion experiments

All results presented in this section relates to the same samples as used in the time resolved photon upconversion experiments presented in Figure 3-6 in the main article.

4.1 Absorption and emission spectrum of TPBAP

Figure S11 and Figure S12 shows absorption and emission spectrum, respectively, of **TPBAP** at different concentrations. The measurements were conducted on the same solutions as was used to prepare the upconversion samples for time resolved upconversion emission presented in the main article.

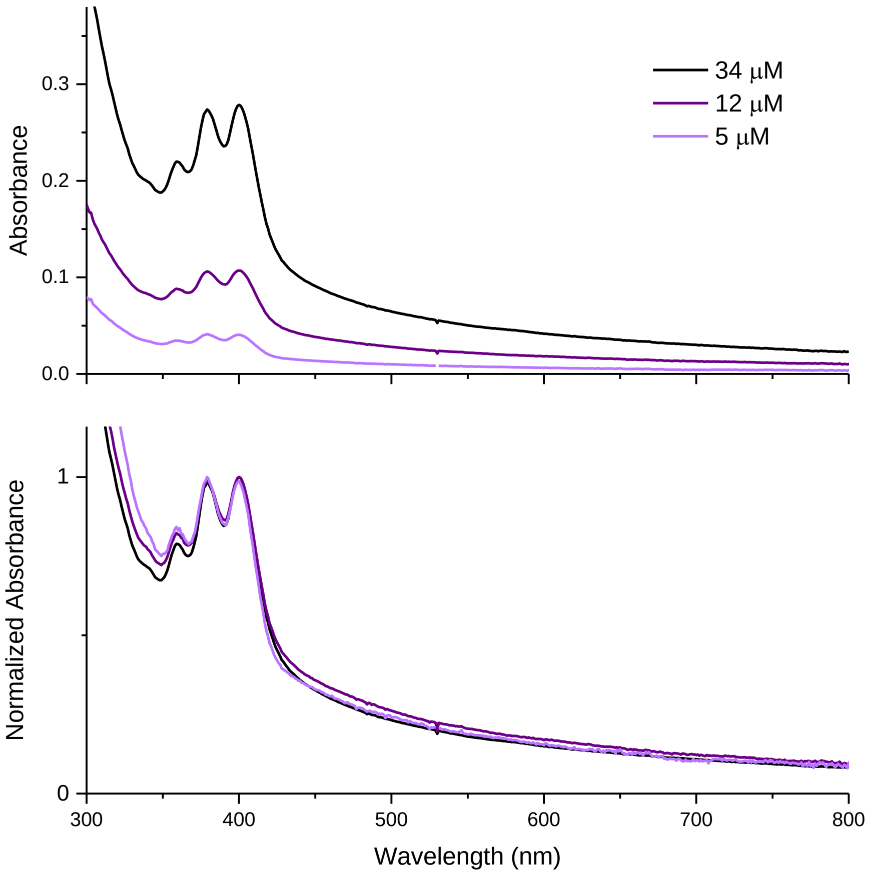

Figure S11. Top: Absorption spectrum of **TPBAP** in THF at different concentration, measured in a 4 mm cuvette. Bottom: Same absorption spectra but normalized at 400 nm.

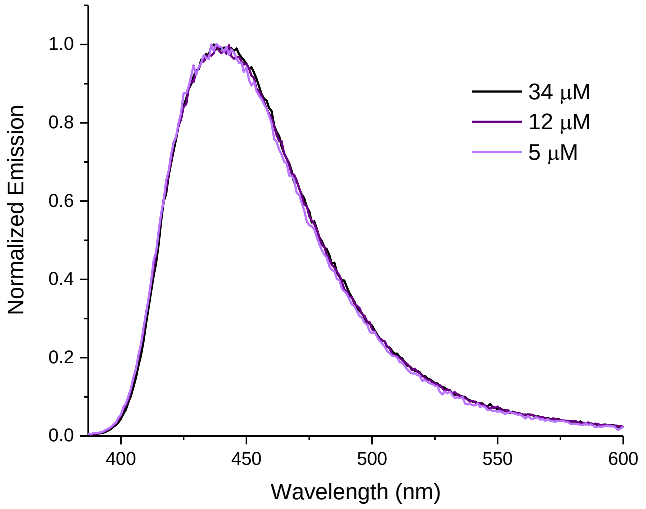

Figure S12. Normalized emission spectra of **TPBAP** in THF at different concentrations. Excitation wavelength 379 nm.

4.2 Time resolved upconversion emission raw data

Figure S13 shows ns time resolved upconversion emission time traces of **TPBAP** and **DPA** at various annihilator concentrations and excitation intensities.

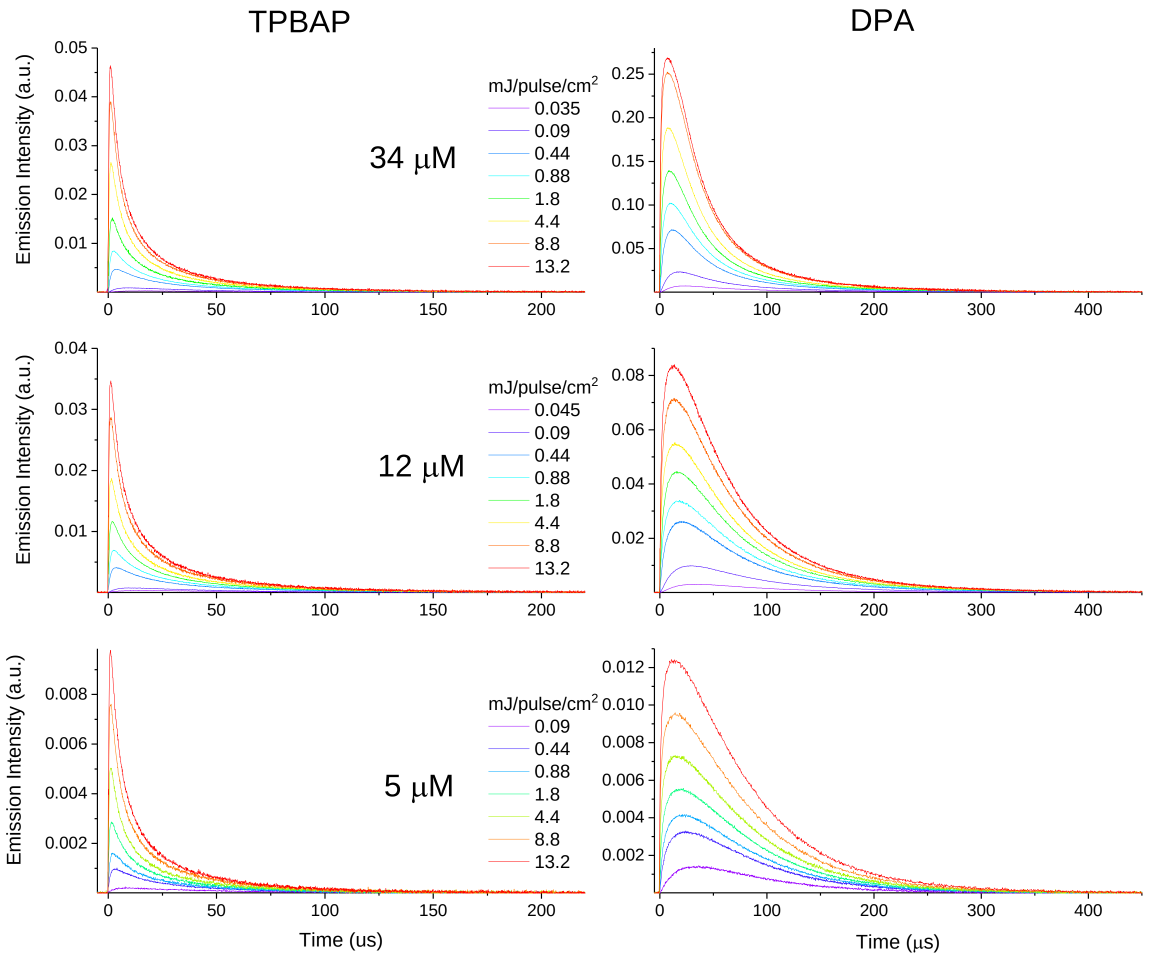

Figure S13. Upconversion emission time traces. Left: **TPBAP+PtOEP**, right: **DPA+PtOEP**. Excitation wavelength 532 nm, emission wavelength centered at 440 nm Annihilator concentration 34 µM (top), 12 µM (middle) and 5 µM (bottom), **PtOEP** concentration 1 mM. Note the different time scales on x-axes

4.3 PtOEP phosphorescence quenching by TPBAP and DPA

Figure S14 shows time traces of **PtOEP** phosphorescence in presence of different concentrations of the annihilators **TPBAP** and **DPA**. The phosphorescence lifetime of **PtOEP** decreases at higher concentrations of **DPA,** indicating triplet sensitization of the annihilator by triplet energy transfer. In contrast, no significant quenching of the **PtOEP** phosphorescence can be seen (within experimental error) in presence of **TPBAP**, indicating very inefficient triplet sensitization of **TPBAP**.

Note that the phosphorescence lifetime of **PtOEP** also without presence of quenching annihilator in this experiment is shorter than its unquenched lifetime due to triplet-triplet

annihilation between the sensitizers. The effect of self-annihilation of **PtOEP** can be seen as excitation intensity dependence of **PtOEP** phosphorescence decay in Figure S15. Unquenched triplet lifetime of **PtOEP** is approximately 100 µs, which can be obtained at low concentration and low excitation intensity, as in Figure S10.

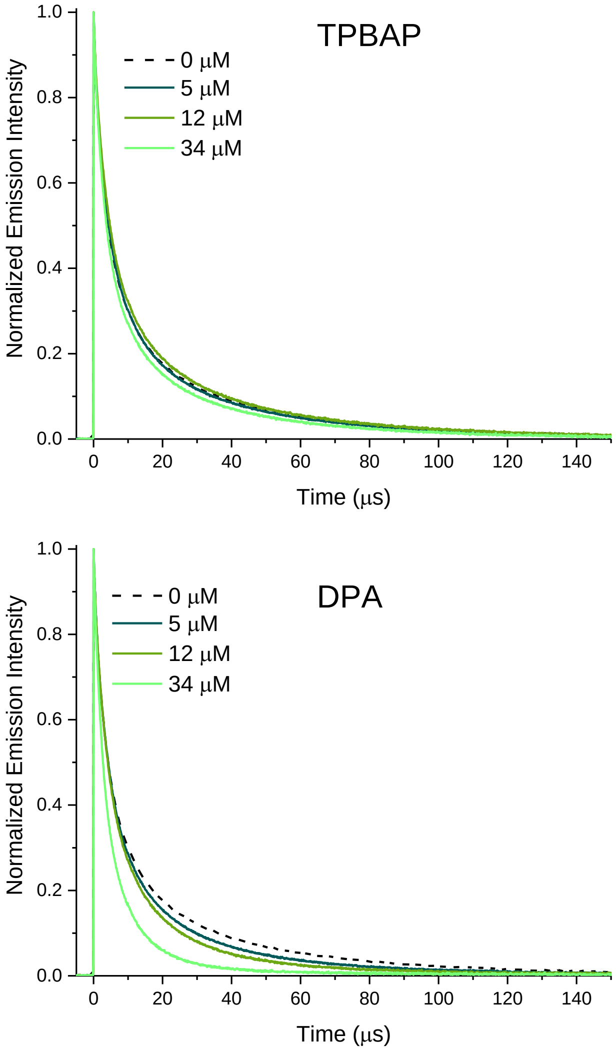

Figure S14. Emission decay of **PtOEP** (1 mM) with and without **TPBAP** and **DPA** annihilator in different concentrations. Excitation wavelength 532 nm, emission wavelength 646 nm, excitation power 0.09 mJ/pulse/cm^2.

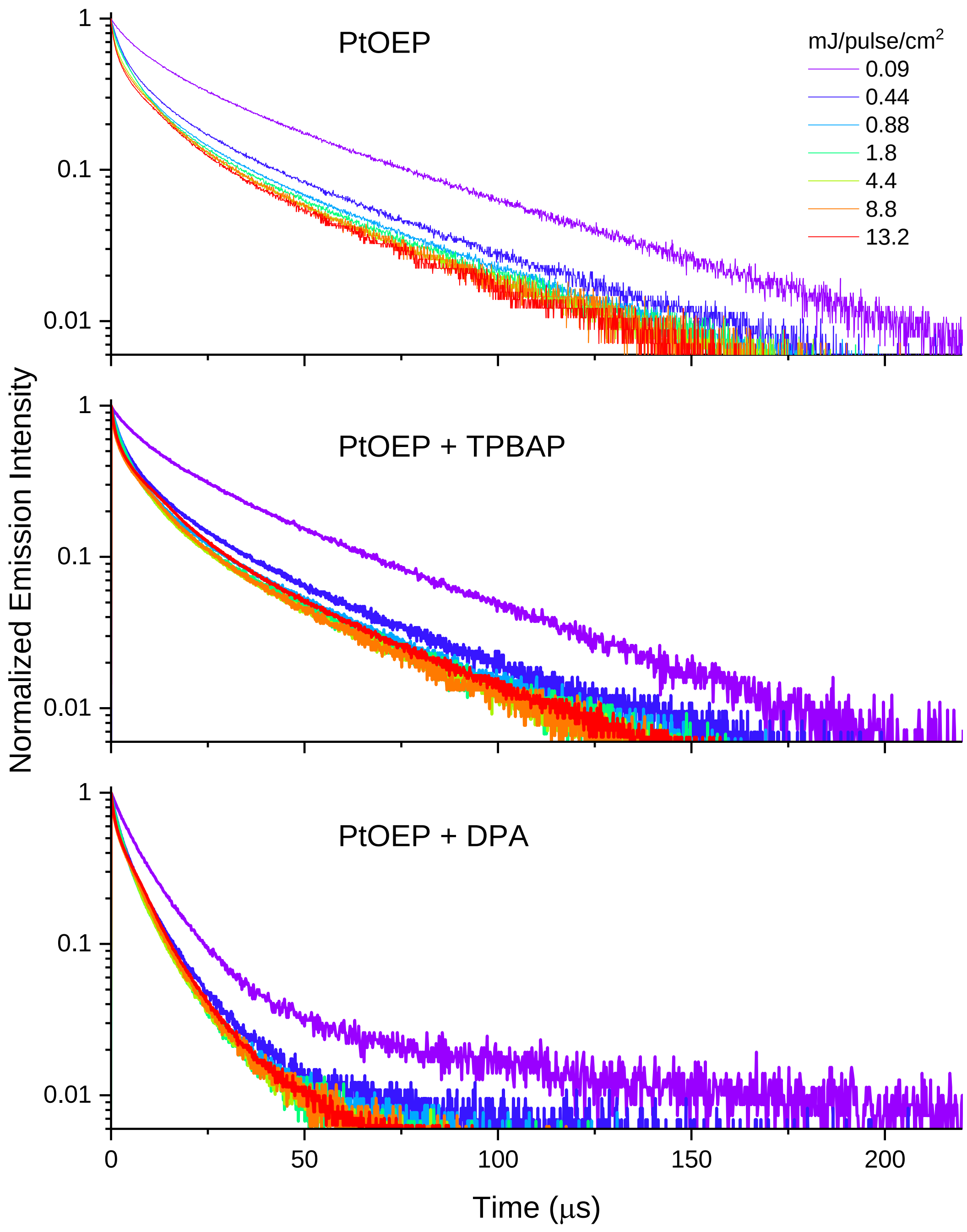

Figure S15. Phosphorescence decay of **PtOEP** (1 mM) at various excitation intensities without and with **TPBAP** and **DPA** annihilator (34 μM). Excitation wavelength 532 nm, emission wavelength 646 nm.

5. Fitting of upconversion time traces

The upconversion time traces in Figure 5 in the main article were fitted to the function in Equation 4 in the main article. The rate constant of spontaneous triplet decay, k_T, were set as a global parameter independent of excitation intensity, while all other parameters, β, α, k_1, k_2, α_1 and α_2 were free fitting parameters for each annihilator and excitation intensity. The fitted parameters can be seen in Table S2.

Table S2. Parameters obtained from fitting of upconversion emission time traces in Figure 5 to Equation 4 in the main article.

**Excitation intensity (mJ/pulse/cm^2)**	k_T **(s^{-1})**	β	α **(a.u.)**	k_1 **(s^{-1})**	k_2 **(s^{-1})**	α_1 **(a.u.)**	α_2 **(a.u.)**
**TPBAP**							
0.09	$6.7\cdot10^3$	0.12	0.058	$6.4\cdot10^3$	$3.8\cdot10^5$	-0.043	-0.010
0.44		0.62	0.053	$9.6\cdot10^3$	$1.2\cdot10^6$	-0.021	-0.021
0.88		0.81	0.055	$2.5\cdot10^4$	$1.6\cdot10^6$	-0.011	-0.031
1.8		0.87	0.084	$4.3\cdot10^4$	$2.2\cdot10^6$	-0.024	-0.040
4.4		0.91	0.118	$5.7\cdot10^4$	$2.9\cdot10^6$	-0.038	-0.048
8.8		0.91	0.143	$5.9\cdot10^4$	$3.1\cdot10^6$	-0.045	-0.056
13.2		0.92	0.162	$6.1\cdot10^4$	$3.2\cdot10^6$	-0.051	-0.063
**DPA**							
0.09	$7.3\cdot10^3$	0.08	0.061	$3.4\cdot10^4$	$1.2\cdot10^5$	-0.029	-0.024
0.44		0.02	0.081	$4.7\cdot10^4$	$3.3\cdot10^5$	-0.033	-0.029
0.88		0.05	0.091	$4.6\cdot10^4$	$4.1\cdot10^5$	-0.035	-0.032
1.8		0.11	0.106	$4.3\cdot10^4$	$4.7\cdot10^5$	-0.039	-0.033
4.4		0.08	0.115	$4.7\cdot10^4$	$5.6\cdot10^5$	-0.036	-0.030
8.8		0.09	0.131	$4.7\cdot10^4$	$6.5\cdot10^5$	-0.039	-0.039
13.2		0.09	0.147	$4.9\cdot10^4$	$7.0\cdot10^5$	-0.042	-0.045

It can be noted that the fitted rate constant of spontaneous triplet decay, k_T, is surprisingly high. It gives a triplet lifetime of $\tau_T = 1/k_T$ of 149 µs and 137 µs for **TPBAP** and **DPA,** respectively. This can be compared to other reported values that are in the range of milliseconds for **DPA**, for example τ_T^{DPA} = 8.61 ms (ref. 8). However, reported values of the triplet lifetime of **DPA** varies significantly, probably because the measured value is very sensitive presence of molecular oxygen, but also because the second order decay channel by TTA is not always taken into account. The reason for the unsuspected short fitted triplet lifetime could be explained by energetically uphill triplet energy back transfer (bTET) from the annihilator back to the **PtOEP** sensitizer.[5,9] This can be checked in the case for **DPA**: The rate constant of triplet energy back transfer, k_{bTET}, is related to the rate of forward TET, k_{TET}, by equation S3

$$k_{bTET} = k_{TET} \cdot e^{-\Delta E/k_B T} \tag{S3}$$

where ΔE is the difference in triplet energy of the donor (in this case **DPA**) and acceptor (in this case **PtOEP**).[10] Triplet energy of **DPA** and **PtOEP** can be estimated to 1.77 eV (ref. 11) and 1.91 eV (ref. 12), respectively, giving ΔE=-0.14 eV. Using k_{TET} = 1.88 $\cdot 10^9$ $M^{-1}s^{-1}$ (ref. 12) and T=295 K yields k_{bTET}=9.6$\cdot 10^6$ $M^{-1}s^{-1}$. This value can be used to calculate the quenched rate constant of triplet decay for **DPA** in presence of **PtOEP** from the Stern-Volmer equation.

$$k_T = 1/\tau_T = 1/\tau_0 + k_{bTET} \cdot [\text{PtOEP}] \qquad \text{(S4)}$$

Using the unquenched triplet lifetime τ_0 = 8.61$\cdot 10^{-3}$ s (ref. 8) and **PtOEP** concentration 1 mM, this gives the quenched rate constant k_T=7.7$\cdot 10^3$ s^{-1}. This is similar to the value of 7.3$\cdot 10^3$ s^{-1} obtained from fitting experimental data in Table S2.

6. Estimating rate constant of iTTA

From Equation 3 in the main article, an apparent rate constant of TTA, k_{TTA} can be calculated as in Equation S4.

$$k_{TTA} = \frac{\beta k_T}{2\,[^3A^*]_{t=0}(1-\beta)} \qquad \text{(S4)}$$

The initial concentration of triplet excited annihilators, $[^3A^*]_{t=0}$, can be estimated form the rate constant of triplet energy transfer, k_{TET}, multiplied with the time dependent concentration of triplet excited sensitizer, $[^3S^*](t)$, and the concentration of ground state annihilator, integrated over a short time, τ.

$$[^3A^*]_{t=0} = \int_0^{\tau} [^3S^*]\,(t) \cdot [^1A\,] \cdot k_{TET}\,dt \qquad \text{(S5)}$$

The rate constant of TET, k_{TET}, for **TPBAP** and **DPA** ($8.2 \cdot 10^7$ $M^{-1}s^{-1}$ and $1.78 \cdot 10^9$ $M^{-1}s^{-1}$, respectively) was obtained from Stern-Volmer quenching experiments, see section 3.6 above and ref. 13, respectively. The integration time, τ, was set to 1 µs, which is an estimate of the time window when most of the TET events from sensitizer to annihilator occurs (which is similar to the rise time of the upconversion signal for **TPBAP**, Figure 3). The initial concentration of triplet excited sensitizer, $[^3S^*](t = 0)$, was set to 10^{-3} M and the concentration time profile, $[^3S^*](t)$, was assumed to follow the measured phosphorescence decay of **PtOEP** in presence of the respective annihilator. Setting the start condition to $[^3S^*](t = 0) = 10^{-3}$ M assumes that all sensitizer molecules in the 10^{-3} M **PtOEP** upconversion sample get excited by the excitation pulse. To justify this, the number of photons from one excitation pulse can be calculated to ~$4 \cdot 10^{15}$ photons per pulse at the highest excitation intensity 13.2 mJ/cm^2/pulse. Because the very high concentration of **PtOEP** and its high molar absorptivity at the excitation wavelength (~50 000 $M^{-1}cm^{-1}$ at 532 nm), it can be assumed that all photons are absorbed within 0.5 mm pathlength into the sample. Given the diameter of the laser (3.8 mm), the excitation volume is $5.6 \cdot 10^{-6}$ dm^3 and the number of **PtOEP** molecules within that volume is $3 \cdot 10^{15}$, which is similar to the number of photons in one excitation pulse. It should be noted that due to absorption saturation at this high excitation intensity, the penetration depth of the excitation light into the sample is longer than what would be expected from the Lambert-Beer law. However, all measurements were done in front face (see section 2 above) which means that the detected emission mainly comes from the excitation region closest to the cuvette edge, where also the excitation photon flux is the highest.

Based on these assumptions, the initial concentration of triplet excited annihilator is calculated from Equation S5 to $3.96 \cdot 10^{-8}$ M and $4.02 \cdot 10^{-8}$ M for **TPBAP** and **DPA** respectively (5 µM annihilator, 13.2 mJ/cm^2/pulse). Using this and values of k_T and β from Table S2, k_{TTA} is calculated using Equation S4 to $9.7 \cdot 10^{11}$ M^{-1}s^{-1} and $9.0 \cdot 10^{9}$ M^{-1}s^{-1} for **TPBAP** and **DPA**, respectively.

References

(1) Schmidt, J.; Weber, J.; Epping, J. D.; Antonietti, M.; Thomas, A. Microporous Conjugated Poly(Thienylene Arylene) Networks. *Adv. Mater.* **2009**, *21*, 702–705.

(2) Bildirir, H.; Osken, I.; Schmidt, J.; Ozturk, T.; Thomas, A. Chemical RedOx Properties of a Donor-Acceptor Conjugated Microporous Dithienothiophene-Benzene Co-Polymer Formed via Suzuki-Miyaura Cross-Coupling. *ChemistrySelect* **2016**, *1*, 748–751.

(3) Thommes, M.; Kaneko, K.; Neimark, A. V; Olivier, J. P.; Rodriguez-Reinoso, F.; Rouquerol, J.; Sing, K. S. W. Physisorption of Gases, with Special Reference to the Evaluation of Surface Area and Pore Size Distribution (IUPAC Technical Report). *Pure Appl. Chem.* **2015**, *87*, 1051–1069.

(4) Crosby, G. A.; Demas, J. N. Measurement of Photoluminescence Quantum Yields. Review. *J. Phys. Chem.* **1971**, *75*, 991–1024.

(5) Gray, V.; Küçüköz, B.; Edhborg, F.; Abrahamsson, M.; Moth-Poulsen, K.; Albinsson, B. Singlet and Triplet Energy Transfer Dynamics in Self-Assembled Axial Porphyrin-Anthracene Complexes: Towards Supra-Molecular Structures for Photon Upconversion. *Phys. Chem. Chem. Phys.* **2018**, *20*, 7549–7558.

(6) Edhborg, F.; Küçüköz, B.; Gray, V.; Albinsson, B. Singlet Energy Transfer in Anthracene–Porphyrin Complexes: Mechanism, Geometry, and Implications for Intramolecular Photon Upconversion. *J. Phys. Chem. B* **2019**, *123*, 9934–9943.

(7) Börjesson, K.; Gilbert, M.; Dzebo, D.; Albinsson, B.; Moth-Poulsen, K. Conjugated Anthracene Dendrimers with Monomer-like Fluorescence. *RSC Adv.* **2014**, *4*, 19846–19850.

(8) Gray, V.; Dzebo, D.; Lundin, A.; Alborzpour, J.; Abrahamsson, M.; Albinsson, B.; Moth-Poulsen, K. Photophysical Characterization of the 9,10-Disubstituted Anthracene Chromophore and Its Applications in Triplet–Triplet Annihilation Photon Upconversion. *J. Mater. Chem. C* **2015**, *3*, 11111–11121.

(9) Isokuortti, J.; Allu, S. R.; Efimov, A.; Vuorimaa-Laukkanen, E.; Tkachenko, N. V; Vinogradov, S. A.; Laaksonen, T.; Durandin, N. A. Endothermic and Exothermic Energy Transfer Made Equally Efficient for Triplet–Triplet Annihilation Upconversion. *J. Phys. Chem. Lett.* **2020**, *11*, 318–324.

(10) Sandros, K. Transfer of Triplet State Energy In Fluid Systems III. Reversile Energy Transfer. *Acta Chemica Scandinavica*. 1964, pp 2355–2374.

(11) Brinen, J. S.; Koren, J. G. The Lowest Triplet State of 9, 10 Diphenylanthracene. *Chem.*

Phys. Lett. **1968**, *2*, 671–672.

(12) Gray, V.; Dreos, A.; Erhart, P.; Albinsson, B.; Moth-Poulsen, K.; Abrahamsson, M. Loss Channels in Triplet-Triplet Annihilation Photon Upconversion: Importance of Annihilator Singlet and Triplet Surface Shapes. *Phys. Chem. Chem. Phys.* **2017**, *19*, 10931–10939.

(13) Olesund, A.; Gray, V.; Mårtensson, J.; Albinsson, B. Diphenylanthracene Dimers for Triplet–Triplet Annihilation Photon Upconversion: Mechanistic Insights for Intramolecular Pathways and the Importance of Molecular Geometry. *J. Am. Chem. Soc.* **2021**, *143*, 5745–5754.
